# Sensory Afferent Renal Nerve Activated Gαi_2_ Subunit Proteins Mediate the Natriuretic, Sympathoinhibitory and Normotensive Responses to Peripheral Sodium Challenges

**DOI:** 10.3389/fphys.2021.771167

**Published:** 2021-11-30

**Authors:** Jesse D. Moreira, Kayla M. Nist, Casey Y. Carmichael, Jill T. Kuwabara, Richard D. Wainford

**Affiliations:** ^1^Whitaker Cardiovascular Institute, School of Medicine, Boston University, Boston, MA, United States; ^2^Department of Medicine, School of Medicine, Boston University, Boston, MA, United States; ^3^Department of Anatomy & Neurobiology, School of Medicine, Boston University, Boston, MA, United States; ^4^Department of Pharmacology and Experimental Therapeutics, School of Medicine, Boston University, Boston, MA, United States

**Keywords:** central Gαi_2_ proteins, afferent renal sympathetic nerves, sodium homeostasis, blood pressure, natriuresis

## Abstract

We have previously reported that brain Gαi_2_ subunit proteins are required to maintain sodium homeostasis and are endogenously upregulated in the hypothalamic paraventricular nucleus (PVN) in response to increased dietary salt intake to maintain a salt resistant phenotype in rats. However, the origin of the signal that drives the endogenous activation and up-regulation of PVN Gαi_2_ subunit protein signal transduction pathways is unknown. By central oligodeoxynucleotide (ODN) administration we show that the pressor responses to central acute administration and central infusion of sodium chloride occur independently of brain Gαi_2_ protein pathways. In response to an acute volume expansion, we demonstrate, via the use of selective afferent renal denervation (ADNX) and anteroventral third ventricle (AV3V) lesions, that the sensory afferent renal nerves, but not the sodium sensitive AV3V region, are mechanistically involved in Gαi_2_ protein mediated natriuresis to an acute volume expansion [peak natriuresis (μeq/min) sham AV3V: 43 ± 4 vs. AV3V 45 ± 4 vs. AV3V + Gαi_2_ ODN 25 ± 4, *p* < 0.05; sham ADNX: 43 ± 4 vs. ADNX 23 ± 6, AV3V + Gαi_2_ ODN 25 ± 3, *p* < 0.05]. Furthermore, in response to chronically elevated dietary sodium intake, endogenous up-regulation of PVN specific Gαi_2_ proteins does not involve the AV3V region and is mediated by the sensory afferent renal nerves to counter the development of the salt sensitivity of blood pressure (MAP [mmHg] 4% NaCl; Sham ADNX 124 ± 4 vs. ADNX 145 ± 4, *p* < 0.05; Sham AV3V 125 ± 4 vs. AV3V 121 ± 5). Additionally, the development of the salt sensitivity of blood pressure following central ODN-mediated Gαi_2_ protein down-regulation occurs independently of the actions of the brain angiotensin II type 1 receptor. Collectively, our data suggest that in response to alterations in whole body sodium the peripheral sensory afferent renal nerves, but not the central AV3V sodium sensitive region, evoke the up-regulation and activation of PVN Gαi_2_ protein gated pathways to maintain a salt resistant phenotype. As such, both the sensory afferent renal nerves and PVN Gαi_2_ protein gated pathways, represent potential targets for the treatment of the salt sensitivity of blood pressure.

## Introduction

Hypertension, or high blood pressure, is the leading risk factor for multiple cardiovascular diseases including stroke, myocardial infarction and chronic kidney disease. Hypertension is estimated to impact 1 in 2 United States adults (Reboussin et al., 2018) and to directly contribute to 10.4 million deaths worldwide per year (Unger et al., 2020). Multiple studies support that excess dietary salt intake increases blood pressure and the risk of premature cardiovascular morbidity and mortality (Appel et al., 2011; Campbell et al., 2011; He et al., 2013; Cook et al., 2014). Despite the mounting evidence of the adverse cardiovascular impact of excess dietary salt approximately 90% of United States adults exceed of the American Heart Association recommended daily intake of <2,300 mmol of sodium for most adults (Lloyd-Jones et al., 2010). The salt sensitivity of blood pressure, an exaggerated blood pressure response to salt intake, is estimated to be present in 50% of hypertensive patients and 25% of normotensive individuals (Kotchen et al., 2013) and increases both hypertension risk and the risk of adverse cardiovascular outcomes (Franco and Oparil, 2006).

It is well established that dietary salt intake can modulate sympathetic nervous system activity and blood pressure through actions in several sites located in the forebrain (Stocker et al., 2013). The circumventricular organs (CVO), particularly the AV3V region which contains the sodium sensitive subfornical organ (SFO) and organum vasculosum of the lamina terminalis (OVLT), play a central role in the actions of salt on sympathetic outflow and blood pressure regulation (Larsen and Mikkelsen, 1995; Stocker et al., 2013; Simmonds et al., 2014). In addition to these central sodium sensing sites, there is increasing evidence that the renal sensory afferent nerves, that project from the renal pelvis to the central nervous system, influence central sympathetic outflow, renal sodium handling and blood pressure in response to perturbations in sodium homeostasis (Kopp, 2015; Frame et al., 2016, 2019).

Despite our understanding that the activation of multiple G-protein coupled receptors (GPCR’s) can evoke sympathoinhibition and natriuresis (e.g., GABA_B_, α_2_-adrenoceptor) there is limited understanding of the downstream signal transduction pathways activated by GPCR’s *in vivo* in response to alterations in sodium homeostasis. Our laboratory has demonstrated a critical role of central Gαi_2_ subunit [guanine nucleotide-binding protein G(i) subunit alpha-2] proteins, which are inhibitory intracellular signaling proteins coupled to GPCRs, in mediating the sympathoinhibitory and normotensive responses to acute peripheral challenges to sodium homeostasis (volume expansion, sodium chloride infusion or sodium chloride bolus administration) in normotensive male Sprague Dawley rats (Kapusta et al., 2012; Wainford et al., 2013; Carmichael et al., 2016, 2020). Significantly, we have reported that dietary sodium-evoked endogenous up-regulation of PVN specific Gαi_2_ subunit proteins represents a conserved mechanism that is required to maintain salt resistance and sympathoinhibition during high dietary salt intake in salt resistant rat phenotypes (Sprague Dawley, Brown Norway and the Dahl Salt Resistant rat) (Kapusta et al., 2013; Wainford et al., 2015; Moreira et al., 2019; Carmichael et al., 2020). In contrast, the Dahl Salt Sensitive rat fails to up-regulate PVN Gαi_2_ proteins during high salt intake and exhibits the attenuated salt sensitivity of blood pressure when the levels of PVN Gαi_2_ proteins are increased (Wainford et al., 2015). Suggesting that Gαi_2_ proteins play a role in blood pressure regulation in human subjects, single nucleotide polymorphisms in the GNAI2 gene associate with hypertension risk and the salt sensitivity of blood pressure in the Genetic Epidemiology of Salt Sensitivity dataset (Zhang et al., 2018).

It remains unknown whether the integrated normotensive and sympathoinhibitory responses to challenges to sodium homeostasis, that are mediated by central Gαi_2_ subunit protein signal transduction pathways, occur in response to changes in sodium detected centrally or peripherally. The current studies were designed to investigate the potential role(s) of central Gαi_2_ proteins in the cardiovascular, sympathoinhibitory and natriuretic responses to central versus peripheral sodium challenges and the influence of established sodium sensing mechanisms on central Gαi_2_ proteins. These studies were conducted in normotensive 3-month-old male Sprague Dawley rats, pre-treated centrally with either a control scrambled (SCR) or targeted Gαi_2_ oligodeoxynucleotide (ODN), prior to undergoing an acute or chronic central versus peripheral sodium loading paradigm.

## Materials and Methods

### Ethical Approval

All animal protocols were approved by the Institutional Animal Care and Use Committee (IACUC) in accordance with the guidelines of Boston University School of Medicine and the National Institutes of Health *Guide for the Care and Use of Laboratory Animals.* As detailed in our surgical procedures, all steps possible were taken to minimize pain and suffering. Additionally, all animal studies detailed in this manuscript fully comply with the ethical principles of Frontiers in Physiology.

### Animals

Three-month-old male Sprague Dawley rats, weighing 275–299 g, were purchased from Envigo (Indianapolis, IN, United States) for use in these studies. Prior to surgical intervention animals were pair-housed and were then housed individually post-surgery. Animals were housed in the Laboratory Animal Science Center at Boston University under a 12 h:12 h light:dark cycle under temperature (20–26°C) and humidity (30–70%) controlled conditions. Animals were provided tap water and standard irradiated rodent normal salt (NS) diet *ad libitum* [Envigo Teklad, WI, Teklad Global Diet #2918: 18% protein, 5% crude fat, 5% fiber, 0.6% K^+^ content, with a total NaCl content of 0.6% (174 mEquiv Na^+^ kg)]. For elevated dietary sodium intake studies, animals were fed an experimental high salt (HS) diet *ad libitum* [Envigo Teklad Diets, WI, TD.03095: 19% protein, 5% crude fat, 3% fiber, 0.8% K^+^ content, with a total NaCl content of 4% [678 m Equiv Na^+^ kg)]. All rats were randomly assigned to experimental groups.

## Surgical Procedures

### Intracerebroventricular (i.c.v.) Cannula Implantation

To enable acute oligodeoxynucleotide (ODN)-mediated down-regulation of central Gαi_2_ proteins animals were anesthetized [ketamine, 30 mg kg intraperitoneally (I.P.) in combination with xylazine, 3 mg kg I.P.] and stereotaxically implanted with a stainless steel cannula (Plastics One Inc., Roanoke, VA, United States) into the right lateral cerebral ventricle (0.3 mm posterior to bregma, 1.3 mm lateral to the midline, and 4.5 mm below the skull surface) (Wainford and Kapusta, 2009, 2010, 2012; Kapusta et al., 2012; Wainford et al., 2013; Carmichael et al., 2016). In all studies i.c.v. cannula implantation occurred at least 5–7 days prior to experimentation.

### Intracerebroventricular (i.c.v.) Oligodeoxynucleotide Infusion

Down-regulation of brain Gαi_2_ protein expression levels in rats was achieved by continuous i.c.v. infusion of a phosphodiesterase oligodeoxynucleotide (ODN) probe that selectively targets Gαi_2_ proteins (5′-CTT GTC GAT CAT CTT AGA-3′) (The Midland Certified Reagent Company Inc., TX, United States). Control studies involved i.c.v. infusion of a scrambled (SCR) ODN (5′-GGG CGA AGT AGG TCT TGG-3′). An NCBI Basic Local Alignment Search Tool search of the *Rattus norvegicus* RefSeq protein database was conducted to confirm the specificity of the Gαi_2_ ODN for the rat Gαi_2_ protein sequence and that the SCR ODN does not match any known rat protein sequence. Multiple publications from our laboratory have confirmed effective (approx. 85%) ODN-mediated down-regulation of Gαi_2_ protein expression and no effect of a control SCR ODN on Gα-subunit protein expression in the acute setting as assessed by Western blotting (Kapusta et al., 2012; Wainford and Kapusta, 2012; Wainford et al., 2013). For chronic ODN infusion, animals were anesthetized (ketamine, 30 mg/kg I.P. in combination with xylazine, 3 mg/kg I.P.) and stereotaxically implanted with a stainless steel cannula into the right lateral cerebral ventricle, which was connected via silastic tubing to a miniosmotic pump (model 2004; Durect Corporation, Cupertino, CA, United States). ODNs were dissolved in isotonic saline and infused i.c.v. at 25 μg/6 μl/day (Kapusta et al., 2013; Moreira et al., 2019; Carmichael et al., 2020).

### Acute Femoral Vein, Femoral Artery and Bladder Cannulation

On the day of the acute study rats were anesthetized with sodium methohexital (20 mg kg I.P., supplemented with 10 mg kg I.V. as required). Rats were instrumented with PE-50 catheters in the left femoral vein and left femoral artery and a PE-240 catheter in the bladder to allow I.V. administration of isotonic saline and experimental sodium challenges, measurement of mean arterial pressure (MAP) and heart rate (HR), and assessment of renal excretory function, respectively (Walsh et al., 2016; Frame et al., 2019; Carmichael et al., 2020; Puleo et al., 2020). Rats were placed in a Plexiglas rat holder and an I.V. infusion of isotonic saline (20 μl min) was maintained during a 2 h surgical recovery period allowing rats to return to full consciousness and renal and cardiovascular function to stabilize prior to study. Mean arterial pressure (MAP) and heart rate (HR) were recorded continuously via the femoral artery cannula using computer-driven BIOPAC data acquisition software (MP150 and AcqKnowledge 3.8.2; BIOPAC Systems Inc., Goleta, CA, United States) connected to an external pressure transducer (P23XL; Viggo Spectramed Inc., Oxnard, CA, United States).

### Radiotelemetry Probe Implantation

Rats were anesthetized with ketamine combined with xylazine (30 mg kg I.P. ketamine, 3 mg kg I.P. xylazine). A radiotelemetry probe (PA-C40, DSI, New Brighton, MN, United States) was implanted into the abdominal aorta via the left femoral artery (Brouwers et al., 2015; Foss et al., 2015; Wainford et al., 2015). All animals underwent surgical recovery for 5–7 days prior to subsequent osmotic minipump implantation.

### Selective Afferent Renal Nerve Ablation

Selective afferent renal nerve ablation (ADNX) was performed via direct application of 33 mM capsaicin to the renal nerves (Foss et al., 2015; Frame et al., 2019). As previously described, under sodium pentobarbital anesthesia (20 mg kg I.P.), each kidney was exposed via a dorsal flank incision and the renal artery and vein were gently separated from the surrounding fascia. Capsaicin (33 mM in isotonic saline containing 5% ethanol and 5% Tween-20) was applied to the isolated renal artery and vein avoiding contact with the surrounding tissue to prevent off-target capsaicin exposure. Any excess capsaicin was dried before suturing the flank muscle and skin. In the sham group, each kidney was exposed and the renal artery and vein were visualized before suturing. A sub-group of ADNX rats underwent an i.c.v. cannula implantation procedure (as described above) 5–7 days post-ADNX surgery to allow an acute i.c.v. Gαi_2_ ODN pre-treatment. The efficacy of selective ADNX was confirmed at the end of the study via (1) ELISA analysis of norepinephrine (NE) content in kidney tissue as per the manufacturer’s instructions (IB89537, IBL America, Minneapolis, MN, United States), and (2) ELISA analysis of renal pelvic calcitonin gene related peptide (CGRP) content as per manufacturer’s instructions (no. 589001, Cayman Chemical Co., Ann Arbor, MI, United States) (Foss et al., 2015; Frame et al., 2019).

### AV3V Lesion

Rats were anesthetized with ketamine combined with xylazine (30 mg kg I.P. ketamine and 3 mg kg I.P. xylazine). An anodal electrolytic lesion (2.5 mA for 25 s) was stereotaxically delivered to the AV3V [stereotaxic coordinates: 0.3 mm posterior to bregma, on midline, 7.5 mm ventral to the midsagittal sinus (Simmonds et al., 2014)] using an insulated 23-g nichrome wire exposed only at the tip. In a separate sham group, the nichrome wire was inserted 4 mm into the brain for 25 s but no lesion was delivered. Immediately following AV3V or sham surgery all rats underwent an i.c.v. cannula implantation procedure (as described above) to allow the pharmacological verification of an AV3V lesion and in certain study groups acute i.c.v. Gαi_2_ ODN pre-treatment. Rats were placed in their home cages and monitored during surgical recovery. The AV3V lesion was verified in all groups by observation of postlesion adipsia assessed as fluid intake less than 5 mL during the first 24 h post-lesion (Callahan et al., 1988) and the absence of a pressor response to i.c.v. angiotensin II (Ang II; 200 ng) (Moreira et al., 2009; Vieira et al., 2013) assessed post-volume expansion study or blood pressure measurement study. Adipsic rats were given 5% sucrose water *ad libitum* to encourage drinking and gradually weaned to normal water over 5-days prior to assignment to an experimental study group.

## Experimental Protocols

### Acute Intracerebroventricular (i.c.v.) Oligodeoxynucleotide Pre-treatment

Twenty-four hours prior to the day of the study, down-regulation of brain Gαi_2_ protein expression levels in rats was achieved by i.c.v. injection (25 μg per 5 μL delivered over 60 s) of a phosphodiesterase ODN probe dissolved in isotonic saline that selectively targets Gαi_2_ proteins (5′-CTT GTC GAT CAT CTT AGA-3′). Control studies involved i.c.v. injection of a SCR ODN (5′-GGG CGA AGT AGG TCT TGG-3′) (The Midland Certified Reagent Company, Midland, TX, United States).

### Acute i.c.v. 1 M NaCl Studies

These studies were conducted in groups of conscious animals pretreated centrally with either a control SCR or targeted Gαi_2_ ODN 24-h prior to study. On the day of study all animals underwent an acute femoral vein and femoral artery cannulation and a 2-h stabilization period, in which rats were infused IV with isotonic saline (20 μl/min) and returned to full consciousness. Following a 2-h recovery and measurement of baseline mean arterial pressure (MAP) over a 30-min period an i.c.v. infusion of 1 M NaCl or 0.9% saline (5 μl over 60 s) (Kinsman et al., 2017a,b) was administered in a randomized order 1 h apart. MAP was recorded continuously via the femoral artery cannula using computer-driven BIOPAC data acquisition software (MP150 and AcqKnowledge 3.8.2; BIOPAC Systems Inc., Goleta, CA, United States) connected to an external pressure transducer (P23XL; Viggo Spectramed Inc., Oxnard, CA, United States) to allow the calculation of the peak ΔMAP (*N* = 6/group). Post-blood pressure recording, animals were euthanized and brains collected and stored at −80°C for analysis of PVN Gαi_2_ protein levels.

### Volume Expansion Studies

Volume expansion studies were conducted on groups of conscious animals maintained on a normal salt diet that had previously undergone ADNX or AV3V surgery and an i.c.v. cannula implantation and subgroups of ADNX and AV3V animals that received an acute i.c.v. Gαi_2_ ODN pre-treatment. After surgical instrumentation and a 2-h stabilization period in which rats were infused IV with isotonic saline (20 μl/min) and returned to full consciousness cardiovascular and renal excretory parameters were measured, and urine was collected during a 20-min control period. The infusate was then increased so that rats received an isotonic saline load equivalent to 5% of body weight (BW) over a 30-min period. Continuous 10-min urine samples were collected during the VE period. The isotonic saline infusate was then returned to a rate of 20 μl/min, after which urine samples were collected during a 90-min recovery period (Wainford and Kapusta, 2010; Kapusta et al., 2012; Carmichael et al., 2016) (*N* = 6 per treatment group). Cardiovascular parameters were continuously recorded during the 120-min experimental protocol. Following completion of the study all animals that received a sham or AV3V lesion received an i.c.v. bolus of Ang II (200 ng/5 μl delivered in 30 s) (Gao et al., 2020) and the peak pressor response over 20 min was recorded. In subgroups of AV3V animals post-fixed brains were sectioned at 20 μm, stained with H&E, and visualized on bright field on a Keyence BZ9000 Microscope for verification of lesion. In all ADNX animals post protocol completion kidneys were collected and stored at −80°C prior to analysis of renal NE content and renal pelvic CGRP levels.

### 14-Day i.c.v. 0.8 M NaCl Infusion Studies

Rats that previously underwent radiotelemetry probe implantation were implanted with osmotic minipumps connected to an i.c.v. cannula to deliver a central infusion of a control SCR or targeted Gαi_2_ ODN (25 μg/6 μl/day). Following a 5-day surgical recovery blood pressure was recorded by radiotelemetry in conscious animals [Dataquest A.R.T. 4.2 software (DSI)] via scheduled sampling for 10 s every 10-min for a 5-day control period (Brouwers et al., 2015; Foss et al., 2015; Wainford et al., 2015). Rats were then randomly assigned to either continue to receive an ODN infusion in 0.9% NaCl or osmotic minipumps were replaced with 0.8 M NaCl (Bunag and Miyajima, 1984; Miyajima and Bunag, 1984) in combination with ODN infusion at 25 μg/day (*N* = 6 per treatment group) and blood pressure was recorded for a further 14-days. Following protocol completion animals were sacrificed by conscious decapitation and plasma was collected for analysis of plasma NE and brains were collected and stored at −80°C for analysis of PVN Gαi_2_ protein levels.

### 21-Day Dietary Sodium Intake Studies

Following ADNX or sham surgery all animals were immediately assigned to receive either 21 days of a normal salt or a high salt diet, *ad libitum*. Following AV3V lesion or sham surgery, and observation of postlesion adipsia during the first 24 h post-lesion, animals were assigned to receive either 21 days of high salt or normal salt diet, *ad libitum* starting 10-days post-lesion. Metabolic balance studies (including assessment of food intake) were conducted on day 20 of salt intake and then animals underwent acute blood pressure measurement on day 21. After surgical instrumentation and a 2-h stabilization period in which rats were infused i.v. with isotonic saline (20 μl/min) and returned to full consciousness, baseline MAP was recorded continuously over a 30-min period in conscious rats (*N* = 6/treatment group/diet). Following completion of the study, all animals that received a sham or AV3V lesion received an i.c.v. bolus of Ang II (200 ng/5 μl delivered in 30 s) (Gao et al., 2020) and the peak pressor response over 20 min was recorded. At the end of protocol, whole brains and both kidneys were collected and stored at −80°C for assessment of PVN Gαi_2_ protein levels and efficacy of ADNX, respectively, as well as assessment of renal norepinephrine content. As previously reported by our laboratory we observed no differences in food intake between the 0.6 and 4% NaCl diet formulations with animals consuming approximately 20 g of food per day.

### Central Losartan Studies

Groups of animals, instrumented with an i.c.v. cannula attached to an osmotic minipumps received either an i.c.v. Gαi_2_ ODN infusion, an i.c.v. losartan (5 μg/hour) (Zimmerman et al., 2004; Wei et al., 2009; Walch et al., 2013) infusion or a Gαi_2_ ODN infusion in combination with losartan. Immediately post cannula implantation animals were randomly assigned to a 21-day normal or high dietary salt intake study. On day 20 all animals underwent a metabolic balance study. On day 21 animals underwent surgical instrumentation and a 2-h stabilization period in which rats were infused i.v. with isotonic saline (20 μl/min) and returned to full consciousness and mean arterial blood pressure was recorded continuously over a 30-min period in conscious rats (*N* = 6/treatment group/diet). After measurement of baseline mean arterial pressure and acute Ang II administration (i.c.v. bolus 200 ng/5 μl delivered in 30 s) (Gao et al., 2020), animals received an acute i.c.v. injection of guanabenz (5 μg/5 μl) and peak changes in heart rate (HR) and MAP were recorded as previously described (*N* = 6/treatment group/diet) (Wainford and Kapusta, 2012; Kapusta et al., 2013).

### Metabolic Balance Studies

Metabolic balance studies were conducted in all groups of rats included in Figures 5, 6. Rats, previously habituated to the metabolic cage for 48 h, were housed in individual metabolic cages (model 18cv, Fenco, MA, United States) with external food containers and water bottles. Metabolic cages were equipped with a double-fine mesh screen that allowed separation of food and feces contamination from urine that was collected in vials containing a layer of mineral oil to prevent evaporation. Rats were randomly assigned to receive a normal or high sodium intake diet and provided access to their respective rodent chow and allowed tap water *ad libitum* via external trays and bottles, respectively. On the day of study, measurements were made for body weight, food and water intake, and urine output during a 24-h period enabling calculation of daily sodium and water balance (Kapusta et al., 2012, 2013; Wainford et al., 2015). Daily sodium balance was determined by calculating the difference between sodium intake (dietary sodium intake) and sodium output (urinary sodium excretion) to enable calculation of whole body sodium retention.

**FIGURE 1:**
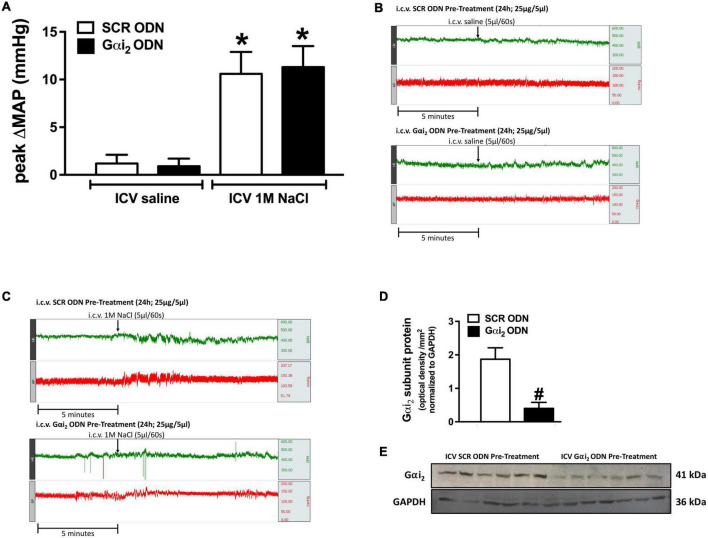
Effect of central Gαi_2_ protein down-regulation on the pressor response to acute central administration of 1 M NaCl. **(A)** Peak change in mean arterial pressure (MAP) in response to the acute i.c.v. administration of 1 M NaCl or 0.9% saline (5 μl over 60 s) in a randomized order 1 h apart in animals pre-treated i.c.v. with an acute SCR or Gαi_2_ ODN injection (25 μg/5 μl) 24-h prior to the day of study, **(B)** representative raw traces of heart rate and blood pressure in conscious male Sprague Dawley rats receiving an acute i.c.v. bolus of 0.9% saline 24-h post an i.c.v. SCR or Gαi_2_ ODN pre-treatment (25 μg/5 μl), **(C)** representative raw traces of heart rate and blood pressure in conscious male Sprague Dawley rats receiving an acute i.c.v. bolus of 1 M NaCl 24-h post an i.c.v. SCR or Gαi_2_ ODN pre-treatment (25 μg/5 μl), **(D)** Gαi_2_-subunit protein expression normalized to GAPDH in the paraventricular nucleus (PVN) of male Sprague Dawley rats that received an i.c.v. SCR or Gαi_2_ ODN pre-treatment (25 μg/5 μl) 24-h prior to the day of study for which the blood pressure responses to i.c.v. 1 M NaCl and 0.9% NaCl are depicted in **(A)**. **(E)** Representative immunoblots of non-pooled PVN samples, loaded equally at a concentration of 20 μg total protein per lane, illustrating hypothalamic Gαi_2_-subunit protein levels in Sprague Dawley rats pre-treated centrally with a SCR or Gαi_2_ ODN. Data are presented as mean ± SEM, *N* = 6/group, *p < 0.05 vs. respective i.c.v. saline group value; ^#^*p* < 0.05 vs. SCR ODN group value.

**FIGURE 2:**
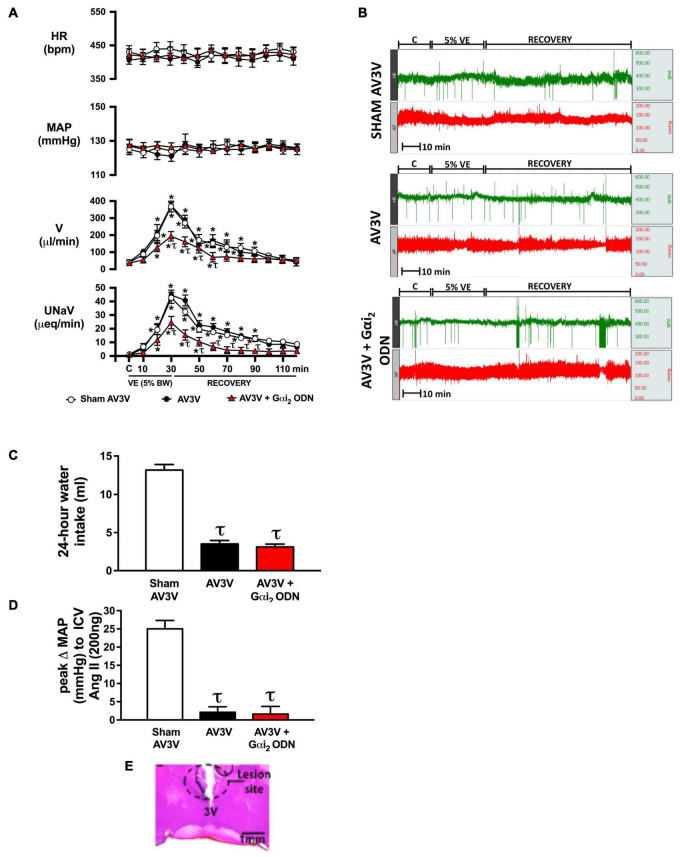
Effect of AV3V lesion alone, and in combination with central Gαi_2_ protein down-regulation, on the cardiovascular and renal excretory responses to an acute volume expansion. **(A)** Cardiovascular and renal responses to a 30-min 5% body weight isotonic saline volume expansion (VE) followed by a 90-min recovery period in conscious male Sprague Dawley rats receiving a sham AV3V lesion, an AV3V lesion or an AV3V lesion and an i.c.v. Gαi_2_ ODN pre-treatment (25 μg/5 μl) 24-h prior to the day of study, **(B)** representative raw traces of heart rate and blood pressure in conscious male Sprague Dawley rats receiving a sham AV3V lesion, an AV3V lesion or an AV3V lesion and an i.c.v. Gαi_2_ ODN pre-treatment (25 μg/5 μl) 24-h prior to the day of study, **(C)** water intake in the 24-h post-AV3V lesion, **(D)** peak change in mean arterial pressure (MAP) to i.c.v. injection of Ang II (200 ng) in sham AV3V, AV3V, and AV3V + Gαi_2_ ODN pre-treated conscious male Sprague Dawley rats, and **(E)** representative image of an AV3V lesion. Data are presented as mean ± SEM, *N* = 6/group. HR, heart rate (bpm); MAP, mean arterial pressure (mmHg); V, urinary flow rate (μL/min); UNaV, urinary sodium excretion (μeq/min). *p < 0.05 vs. respective group baseline control value (denoted C); ^τ^*p* < 0.05 vs. respective sham AV3V group value.

**FIGURE 3:**
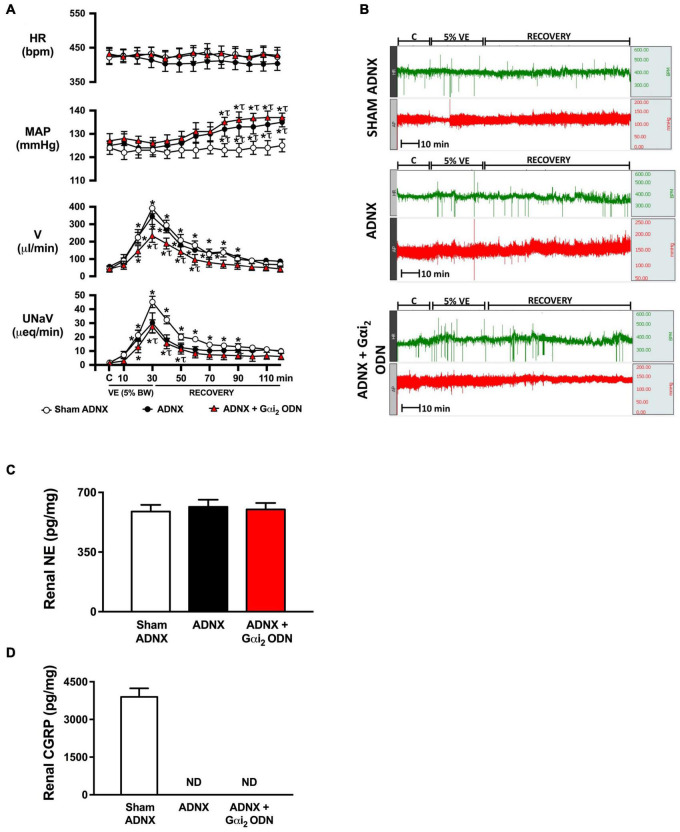
Effect of afferent renal denervation (ADNX) alone, and in combination with central Gαi_2_ protein down-regulation, on the cardiovascular and renal excretory responses to an acute volume expansion. **(A)** Cardiovascular and renal responses to a 30-min 5% body weight isotonic saline volume expansion (VE) followed by a 90-min recovery period in conscious male Sprague Dawley rats receiving a sham ADNX, ADNX, or ADNX and an i.c.v. Gαi_2_ ODN pre-treatment (25 μg/5 μl) 24-h prior to the day of study, **(B)** representative raw traces of heart rate and blood pressure in conscious male Sprague Dawley rats receiving a sham ADNX, ADNX, or ADNX and an i.c.v. Gαi_2_ ODN pre-treatment (25 μg/5 μl) 24-h prior to the day of study, **(C)** renal norepinephrine content (pg/mg), and **(D)** renal pelvic CGRP content (pg/mg). Data are presented as mean ± SEM, *N* = 6/group. HR, heart rate (bpm); MAP, mean arterial pressure (mmHg); V, urinary flow rate (μL/min); UNaV, urinary sodium excretion (μeq/min). ND, not detectable. *p < 0.05 vs. respective group baseline control value (denoted C). ^τ^*p* < 0.05 vs. respective sham ADNX group value.

**FIGURE 4:**
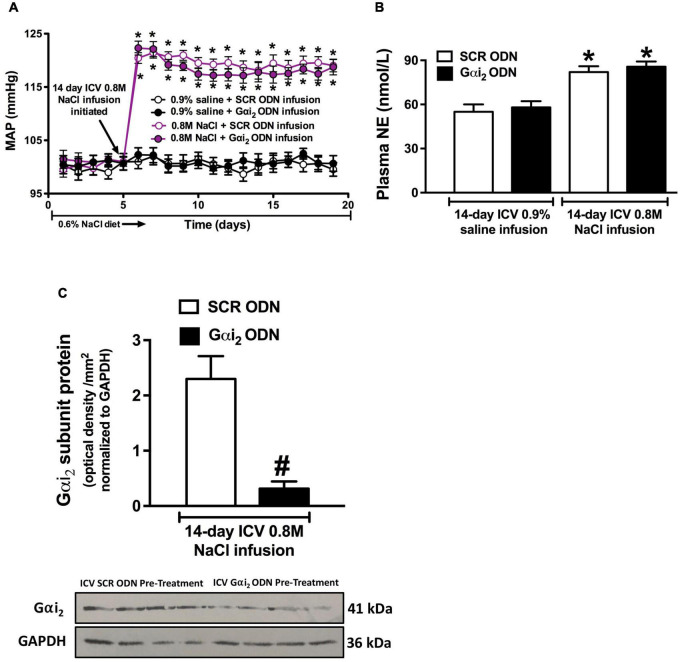
Effect of central Gαi_2_ protein down-regulation on the blood pressure and sympathetic response to central infusion of 0.8 M NaCl. **(A)** Blood pressure response to i.c.v. infusion of 0.8 M NaCl or 0.9% saline in conscious male Sprague Dawley receiving an i.c.v. co-infusion of a SCR or Gαi_2_ ODN injection (25 μg/6 μl/day), **(B)** renal norepinephrine content (pg/mg), and **(C)** Gαi_2_-subunit protein expression of non-pooled PVN samples, loaded equally at a concentration of 20 μg total protein per lane, normalized to GAPDH in the paraventricular nucleus (PVN) of male Sprague Dawley rats that received an i.c.v., SCR or Gαi_2_ ODN infusion (25 μg/6 μl/day) in combination with 0.8 M NaCl for which the blood pressure response is depicted in **(A)**. Data are presented as mean ± SEM, *N* = 6/group, *p < 0.05 vs. respective i.c.v. saline group value; ^#^*p* < 0.05 vs. SCR ODN group value.

**FIGURE 5:**
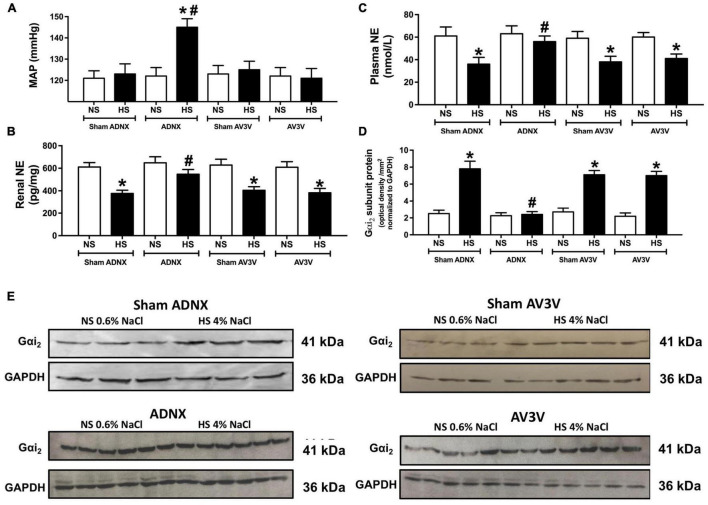
Effect of an AV3V lesion or ADNX on PVN Gαi_2_ protein expression and the salt sensitivity of blood pressure. **(A)** MAP (mmHg), **(B)** renal norepinephrine (NE) content (pg/mg) **(C)** plasma NE content (nmol/L), **(D)** Gαi_2_-subunit protein expression of non-pooled PVN samples normalized to GAPDH in the paraventricular nucleus (PVN) of male Sprague Dawley rats that underwent an AV3V lesion or ADNX, and **(E)** representative immunoblots of non-pooled PVN samples, loaded equally at a concentration of 20 μg total protein per lane in male Sprague Dawley rats that underwent an AV3V lesion or ADNX that were maintained on a normal or high salt-intake for 21-days. Data are presented as mean ± SEM, *N* = 6/group. *p < 0.05 vs. respective normal salt-intake group value. ^#^*p* < 0.05 vs. sham ADNX high salt-intake group value.

**FIGURE 6:**
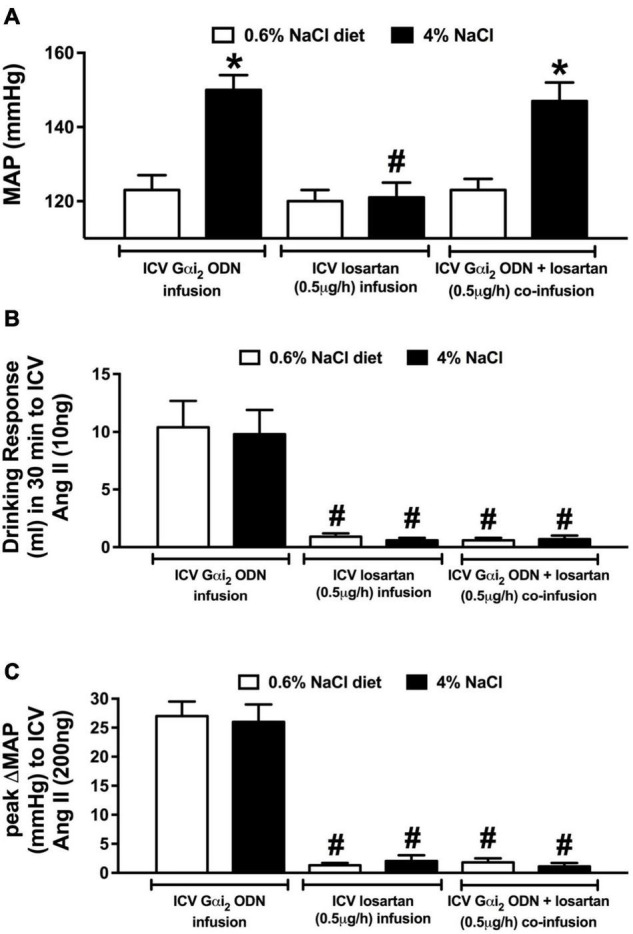
Effect of brain AT1R antagonism on the salt sensitivity of blood pressure in central Gαi_2_ ODN infused rats. **(A)** MAP (mmHg), **(B)** drinking response (ml) in 30 min to i.c.v. Ang II (10 ng), and **(C)** peak change in mean arterial pressure (MAP) to i.c.v. injection of Ang II (200 ng) in conscious male Sprague Dawley rats receiving a central Gαi_2_ ODN infusion (25 μg/6 μl/day), a central losartan infusion (0.5 μg/h) or a central Gαi_2_ ODN:losartan co-infusion (25 μg/6 μl/day) for 21-days that were maintained on a normal or high salt-intake. Data are presented as mean ± SEM, *N* = 6/group. *p < 0.05 vs. respective normal salt-intake group value. ^#^*p* < 0.05 vs. respective i.c.v. Gαi_2_ ODN infusion group value.

### Measurement of Brain Gα-Subunit Protein Levels

Following completion of certain experimental protocols, whole brains were removed and frozen at −80°C. Hypothalamic paraventricular nucleus (PVN) samples were extracted from frozen brains cut on a cryostat using a brain punch tool (Stoelting, IL, United States) as previously described (Kapusta et al., 2012, 2013). Tissue lysates were prepared from individual brain punch samples (i.e., samples were not pooled from multiple animals) and protein levels were quantified using a BCA assay as per manufacturers’ instruction (Thermo Scientific, IL, United States). Individual non-pooled tissue lysates were loaded at a concentration of 20 μg total protein and were resolved on a 10% SDS-PAGE gel and transferred to nitrocellulose membrane (GE Healthcare, NJ, United States). Gαi_2_ levels were determined as previously published by our laboratory (Kapusta et al., 2012, 2013) using a commercially available primary antibody purchased from Santa Cruz Biotechnologies (Santa Cruz, CA, United States), directed against Gαi_2_ (1:200, sc-13534, RRID:AB_627644) and protein levels were normalized to GAPDH (anti-GAPDH 1:1000, ab-9483, Abcam, MA, RRID:AB_307273). In all cases, blots were exposed to a horseradish peroxidase-conjugated secondary antibody (Invitrogen, Thermo Fisher, catalog no. 62-6520, Waltham, MA, United States; RRID:AB_2533947). Chemiluminescent immunoreactive bands were detected by Amersham™ ECL™ Prime western blotting detection reagent (GE Healthcare, RPN2232) and data was imaged and semi-quantified using Bio-Rad Quantity One image analysis software (Kapusta et al., 2012; Wainford et al., 2015; Moreira et al., 2019).

## Analytical Techniques

### Analysis of Urine

Urine volume was determined gravimetrically assuming 1 g = 1 ml. Urinary and plasma sodium concentrations were measured by flame photometry (model 943, Instrumentation Laboratories, MA, United States) (Kapusta et al., 2013; Wainford et al., 2013, 2015).

### Analysis of Plasma

Plasma norepinephrine (NE) levels were determined as previously described (Kapusta et al., 2013; Wainford et al., 2013, 2015). In brief, following plasma collection samples were frozen and stored at −80°C for later analysis. Plasma NE levels were quantified using an ELISA kit (Immuno-Biological Laboratories, Inc., Minneapolis, MN, United States; cat#IB89552) as per manufacturers’ instructions (Wainford et al., 2015; Frame et al., 2019; Moreira et al., 2019).

### Analysis of Renal Norepinephrine Content

Kidneys were harvested from rats following completion of the experimental protocol, flash frozen and stored at −80°C. NE content of kidney was determined with an ELISA (Immuno-Biological Laboratories, Inc., Minneapolis, MN, United States; cat#IB89537) as per manufacturers’ instructions.

### Analysis of Renal Pelvic Calcitonin Gene Related Peptide Content

Kidneys were harvested from rats following completion of the experimental protocol, and the renal pelvis was extracted, flash frozen and stored at −80°C. Renal pelvic GCRP content was determined with an ELISA (no. 589001, Cayman Chemical Co., Ann Arbor, MI, United States) as per manufacturers’ instructions (Foss et al., 2015; Frame et al., 2019).

## Statistical Analysis

All data are expressed as mean ± SEM. Peak change in MAP and HR, plasma NE content, 24-h sodium and water balance and Gαi_2_ protein levels were compared with respective group control values by a student’s *t*-test. Differences occurring between treatment groups were assessed by a two-way ANOVA with a subsequent Sidak’s multiple comparisons test. The magnitude of change in cardiovascular and renal excretion parameters at different time points after acute volume expansion were compared with respective group control values by a one-way repeated-measures (RM) ANOVA with a subsequent Dunnett’s multiple comparisons test. Differences occurring between treatment groups were assessed by a two-way RM (mixed-model) ANOVA (treatment × time) with a subsequent Sidak’s multiple comparisons test. All statistical analyses were performed using Graphpad (GraphPad Prism v.8 for Mac OS X, GraphPad Software, San Diego, CA, United States). In each case, statistical significance was defined as *p* < 0.05.

## Results

### Acute i.c.v. 1 M NaCl Evokes a Brain Gαi_2_ Protein-Independent Response

In all animals i.c.v. saline administration, either pre- or post i.c.v. 1 M NaCl did not alter blood pressure in conscious rats (baseline MAP [mmHg]; SCR ODN 124 ± 4, Gαi_2_ ODN 126 ± 5). In contrast i.c.v. 1 M NaCl evoked a significant acute pressor response in both SCR and Gαi_2_ ODN pre-treated conscious male rats, the magnitude of which was not altered by ODN-mediated down-regulation of central Gαi_2_ proteins (peak ΔMAP [mmHg]; SCR ODN + 10.6 ± 2.3 vs. Gαi_2_ ODN + 11.3 ± 2.2, Figures 1A–C). The efficacy of central Gαi_2_ ODN pre-treatment was confirmed by the observation of a significant reduction (approximately 80%) in the expression of PVN Gαi_2_ proteins (Figures 1D,E).

### Brain Gαi_2_ Protein-Dependent Responses to an Acute i.v. Volume Expansion Occur Independent of the AV3V Region

An acute 5% BW volume expansion did not alter MAP or HR at any time point in sham AV3V lesioned conscious rats (Figures 2A,B). In sham AV3V rats, replicating our prior published data in male Sprague Dawley rats an acute 5% BW volume expansion evoked profound natriuresis and diuresis (Figure 2A). In conscious animals that underwent an AV3V lesion the response to an acute 5% volume expansion was indistinguishable from that observed in sham AV3V lesioned animals. In contrast, in conscious animals that underwent an AV3V lesion in combination with a 24-h Gαi_2_ ODN pre-treatment we observed profoundly attenuated natriuretic and diuretic responses to a 5% BW volume expansion [peak V (μL/min) sham: 368 ± 32 vs. AV3V 374 ± 18 vs. AV3V + Gαi_2_ ODN 193 ± 18, *p* < 0.05; peak UNaV (μeq/min) sham: 43 ± 4 vs. AV3V 45 ± 4 vs. AV3V + Gαi_2_ ODN 25 ± 4, *p* < 0.05] with no change in cardiovascular parameters throughout the protocol (Figures 2A,B).

To confirm the efficacy of AV3V lesions all rats underwent confirmation of post-lesion adipsia (Figure 2C). Following completion of the acute volume expansion protocol all animals received an acute i.c.v. bolus injection of Ang II (200 ng). In sham animals we observed a pressor response to Ang II which was absent in all AV3V lesioned rats (Figure 2D). AV3V lesions were also confirmed histologically (Figure 2E).

### Brain Gαi_2_ Protein-Dependent Responses to an Acute i.v. Volume Expansion Involve the Afferent Renal Nerves

Replicating our prior published data in conscious male Sprague Dawley rats an acute 5% BW volume expansion did not alter MAP or HR, but evoked a profound diuretic and natriuretic response in sham ADNX rats (Figures 3A,B). In animals that underwent ADNX surgery we observed a profound volume expansion-evoked diuretic response of the same magnitude as seen in sham ADNX animals with no change in HR. In contrast, a 5% BW volume expansion in conscious ADNX animals evoked a significant increase in MAP and profoundly attenuated the natriuretic response [peak MAP (mmHg) sham: 125 ± 3 vs. ADNX 135 ± 2, *p* < 0.05; peak UNaV (μeq/min) sham: 43 ± 4 vs. ADNX 23 ± 6, *p* < 0.05] (Figures 3A,B). In conscious animals that underwent ADNX in combination with a 24-h Gαi_2_ ODN pre-treatment, the physiological blood pressure and natriuretic responses to an acute 5% volume expansion were indistinguishable from those observed in ADNX animals with the addition of a profoundly attenuated diuretic response.

To confirm the efficacy of ADNX all rats underwent analysis of renal NE and renal pelvic CGRP. The surgical procedure of ADNX did not alter renal NE levels vs. sham animals and reduced renal pelvic CGRP content to undetectable levels (Figures 3C,D).

### Chronic i.c.v. 0.8 M NaCl Evokes a Brain Gαi_2_ Protein-Independent Response

A 14-day i.c.v. saline infusion, in combination with either a SCR or Gαi_2_ ODN did not alter blood pressure or plasma NE levels (Figures 4A,B). In contrast, i.c.v. 0.8 M NaCl infusion evoked a significant rapid and persistent significant elevation in MAP in both SCR and Gαi_2_ ODN co-infused conscious rats that was accompanied by an increase in plasma NE levels, the magnitude of which was not altered by ODN-mediated down-regulation of central Gαi_2_ proteins (Figures 4A,B). The efficacy of chronic central Gαi_2_ ODN pre-treatment was confirmed by the observation of a significant reduction (approximately 85%) in the expression of PVN Gαi_2_ proteins (Figure 4C).

### Brain Gαi_2_ Protein-Dependent Responses to Dietary Salt Intake Involve the Afferent Renal Nerves

In conscious sham AV3V, sham ADNX and AV3V lesioned rats, in which the afferent renal nerves are intact, a 21-day high salt intake did not alter MAP or sodium balance and evoked global and renal sympathoinhibition that was accompanied by the up-regulation of PVN Gαi_2_ protein levels (Figure 5 and Table 1). In contrast, we observed the development of sodium retention, sympathoexcitation and the salt sensitivity of blood pressure in conscious ADNX animals (Figure 5 and Table 1). Critically, in ADNX rats, which developed the salt sensitivity of blood pressure, we did not observe dietary sodium-evoked up-regulation of PVN Gαi_2_ proteins (Figure 5). The efficacy of AV3V lesions was confirmed by the observation of post-lesion adipsia and the absence of a pressor response to an acute i.c.v. bolus injection of Ang II (200 ng) (Table 1). Confirming the efficacy of ADNX on day 21 days post-ADNX renal pelvic CGRP levels were less than 90% of sham animals (Table 1).

**TABLE 1:** 24-h sodium balance (meq), Renal pelvic CGRP content (pg/mg), 24-h water intake (ml) post lesion and peak ΔMAP (mmHg) post-i.c.v. Ang II (200 ng) in male Sprague Dawley rats that underwent a sham surgery, ADNX surgery or AV3V lesion prior to 21-days normal or high salt sodium diet.

	Sham ADNX		ADNX		Sham AV3V		AV3V	
Parameter	0.6% NaCl	4% NaCl	0.6% NaCl	4% NaCl	0.6% NaCl	4% NaCl	0.6% NaCl	4% NaCl
24 h Na^+^ balance (meq)	0.33 ± 0.1	0.40 ± 0.38	0.41 ± 0.2	2.89 ± 0.4*^#^	0.38 ± 0.2	0.34 ± 0.2	0.29 ± 0.1	0.31 ± 0.1
Renal pelvic CGRP (pg/mg)	4103 ± 308	3962 ± 344	362 ± 58^#^	389 ± 46^#^	–	–	–	–
24 h water intake (ml) post lesion	–	–	–	–	12.4 ± 1.4	13.2 ± 1.6	4.5 ± 1^#^	3.9 ± 0.8^#^
Peak ΔMAP (mmHg) to i.c.v. Ang II 200 ng	–	–	–	–	22 ± 2.3	24 ± 1.4	1.5 ± 1.2^#^	0.9 ± 1.4^#^

The values are the means ± SEM (N = 6 per group). *p < 0.05 compared with respective normal sodium intake group value. ^#^p < 0.05 compared with respective sham group value.

### Central Gαi_2_ Oligodeoxynucleotide-Mediated Salt Sensitivity of Blood Pressure Occurs Independently of Brain Angiotensin II Type 1 Receptor (AT1R) Signaling

Replicating our prior data (Figure 5) a central Gαi_2_ ODN infusion evoked the development of the salt sensitivity of blood pressure following 7-days HS intake that was accompanied by fluid and sodium retention (Figure 6A and Table 2). Central losartan infusion did not impact blood pressure in animals maintained on a normal or high salt intake. Further, central losartan co-infusion had no effect on the magnitude of the salt sensitivity of blood pressure, fluid retention or sodium retention in animals receiving an i.c.v. Gαi_2_ ODN infusion (MAP [mmHg]; Gαi_2_ ODN HS 150 ± 4 vs. Losartan + Gαi_2_ ODN HS 147 ± 5, Figure 6A and Table 2). In these animals the efficacy of AT1R antagonism was confirmed by the absence of a pressor and dipsogenic response to i.c.v. Ang II administration (Figures 6B,C). The efficacy of central Gαi_2_ protein down-regulation was confirmed pharmacologically (Kapusta et al., 2012; Wainford and Kapusta, 2012) by the observation of a bradycardic response to i.c.v. guanabenz in the absence of a hypotensive response (Table 2).

**TABLE 2:** 24-h water balance (ml), 24-h sodium balance (meq), peak ΔHR (bpm) post-i.c.v. guanabenz (5 μg) and peak ΔMAP (mmHg) post-i.c.v. guanabenz (5 μg) in male Sprague Dawley rats receiving either an i.c.v. infusion of a Gαi_2_ ODN (25 μg/6 μl/day), an i.c.v. infusion of losartan (12 μg/6 μl/day – 0.5 μg/hour), or an i.c.v. co-infusion of a Gαi_2_ ODN (25 μg/day) and losartan (12 μg/day) measured on day 7 of a normal or high salt sodium diet.

	i.c.v. Gαi_2_ ODN infusion		i.c.v. losartan infusion		i.c.v. losartan and Gαi_2_ ODN co-infusion	
Parameter	0.6% NaCl	4% NaCl	0.6% NaCl	4% NaCl	0.6% NaCl	4% NaCl
24 h H_2_0 balance (ml)	12.4 ± 1.1	19.9 ± 2.8*	10.8 ± 1.4	12.7 ± 2.1	12.8 ± 1.5	22.4 ± 3.9*^#^
24 h Na^+^ balance (meq)	0.36 ± 0.18	2.62 ± 0.28*	0.45 ± 0.21	0.39 ± 0.19	0.42 ± 0.18	2.34 ± 0.22*^#^
Peak ΔHR (bpm) to i.c.v. guanabenz	−85 ± 8	−92 ± 6	−73 ± 8	−86 ± 9	−81 ± 7	−77 ± 7
Peak ΔMAP (mmHg) to i.c.v. guanabenz	−2.1 ± 1.4^#^	1.3 ± 1.2^#^	−22.4 ± 2.6	−20 ± 3.1	−1.6 ± 1.4^#^	−2.0 ± 1.8^#^

The values are the means ± SEM (N = 6 per group). *p < 0.05 compared with respective normal sodium intake group value. ^#^p < 0.05 compared with respective i.c.v. losartan infused group value.

## Discussion

These studies were designed to determine the potential role(s) of central Gαi_2_ proteins in the integrated cardiovascular, sympathoinhibitory and natriuretic responses to central versus peripheral sodium challenges and the role(s) of established sodium sensing mechanisms on central Gαi_2_ protein-mediated responses and expression. Our studies demonstrate that the acute and chronic central administration of NaCl evokes a pressor response that is independent from brain Gαi_2_ subunit protein signal transduction pathways. Second, we demonstrate that the sensory afferent renal nerves are required to facilitate dietary sodium-evoked PVN Gαi_2_ protein up-regulation, natriuresis and normotension following chronic high dietary sodium intake. Thirdly, we provide evidence that the salt sensitivity of blood pressure that develops following the down-regulation of central Gαi_2_ proteins does not involve a brain AT1R signal transduction mechanism. Collectively our data suggest that central Gαi_2_ proteins are essential in mediating the integrated cardiovascular and renal responses to peripheral, but not central, alterations in sodium homeostasis. Further, the maintenance of salt resistance, which involves PVN specific up-regulation of PVN Gαi_2_ proteins is driven by the sensory afferent renal nerves.

### Central NaCl Evokes a Brain Gαi_2_ Protein-Independent Pressor Response

**FIGURE 7:**
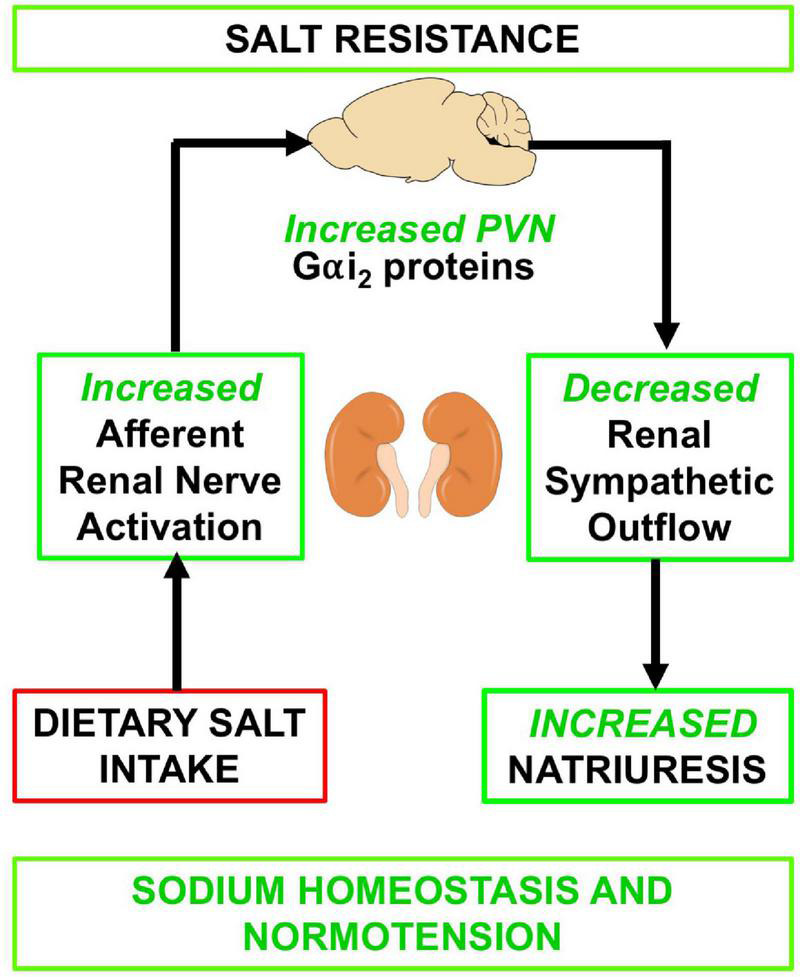
Schematic of proposed mechanism by which the sensory afferent renal nerves influence PVN Gαi_2_ subunit proteins in response to dietary salt intake.

Several central sites, including the SFO and OVLT, (Larsen and Mikkelsen, 1995; Stocker et al., 2013; Simmonds et al., 2014) play an important role in mediating the actions of NaCl on blood pressure, in part through these areas’ connections with the hypothalamic PVN, an integrative neural control center that modulates blood pressure and sympathetic outflow (Stocker et al., 2013; Fujita and Fujita, 2016). Our prior studies have shown a key role of central, and PVN specific, Gαi_2_ proteins in influencing the cardiovascular, renal and sympathetic nervous system responses to acute challenges to sodium homeostasis and chronic elevations in dietary sodium intake (Figure 7; Kapusta et al., 2012, 2013; Wainford et al., 2013; Moreira et al., 2019; Carmichael et al., 2020). However, the impact of central Gαi_2_ proteins on the blood pressure responses to direct increases in central NaCl remain unknown. In these studies, we utilized two different central NaCl challenges: an acute i.c.v. 1 M NaCl bolus (Kinsman et al., 2017a,b) and a chronic 14-day 0.8 M i.c.v. NaCl infusion (Bunag and Miyajima, 1984; Miyajima and Bunag, 1984). In response to both an acute i.c.v. 1 M NaCl bolus injection and a 0.8 M NaCl i.c.v. infusion we observed a significant increase in blood pressure that was not impacted by Gαi_2_ protein down-regulation. The down-regulation of Gαi_2_ proteins by acute ODN pre-treatment and continuous ODN infusion was confirmed by immunoblotting of the PVN as previously reported by our laboratory (Kapusta et al., 2012, 2013; Wainford et al., 2013; Moreira et al., 2019; Carmichael et al., 2020). These results suggest that central administration of NaCl, which evokes a pressor response, does so via mechanisms independent of brain Gαi_2_ protein-gated pathways.

### Acute Volume Expansion Evokes an Afferent Renal Nerve and Gαi_2_ Protein-Independent Natriuretic Response

An acute isotonic volume expansion is a well-established sympathoinhibitory challenge that evokes profound diuresis and natriuresis, partly via suppression of renal sympathetic outflow, to maintain fluid and electrolyte balance and normotension. We have previously demonstrated that brain, and PVN specific, Gαi_2_ subunit protein gated-signal transduction pathways mediate the full natriuretic and diuretic responses to an i.v. volume expansion (Kapusta et al., 2012; Carmichael et al., 2020). These studies were designed to investigate the association between central Gαi_2_ proteins and the sodium/osmo-sensitive AV3V region as well as the sensory afferent renal nerves during an acute volume expansion. Our data, which show that validated AV3V lesions (lesion validated physiologically, pharmacologically, and histologically), had no impact on the cardiovascular and renal responses to an acute volume expansion or the ability of Gαi_2_ down-regulation to attenuate volume expansion evoked natriuretic and diuretic responses. This suggests that the AV3V sodium sensitive region has no role in the acute Gαi_2_ protein-mediated physiological responses to an acute volume expansion.

In contrast, our data show that validated ADNX (ADNX validated by absence of renal pelvic CGRP and the presence of unchanged renal NE levels) attenuates volume expansion-evoked natriuresis and results in an increase in blood pressure – data that replicates our prior findings (Frame et al., 2019). In animals in which Gαi_2_ proteins are downregulated, ADNX is shown not to impact Gαi_2_ ODN-mediated reductions in diuresis or natriuresis and evokes increased blood pressure in response to a volume expansion. Collectively these data suggest that the renal sensory afferent nerves and not the central sodium sensing AV3V region mediate the integrated physiological responses to an acute volume expansion – potentially by mediating a sympathoinhibitory reno-renal reflex to mediate suppression of renal sympathetic nerve traffic and subsequent natriuresis (Kopp et al., 1987; Kopp, 2015; Frame et al., 2019). Further our data suggests potential cross talk between the renal sensory afferent nerves and central Gαi_2_ subunit protein-gated pathways to mediate the natriuretic, but not diuretic or blood pressure, response to perturbations in whole body sodium homeostasis evoked by an acute volume expansion. In these animals which underwent an acute volume expansion, we did not validate Gαi_2_ protein down-regulation by immunoblotting – however, given that in all animals which received a Gαi_2_ ODN we observed the same pattern and magnitude of attenuated natriuresis and diuresis as we have previously published, (Kapusta et al., 2012; Carmichael et al., 2020) we are confident in the efficacy of our approach.

### The Sensory Afferent Renal Nerves Are Required for Dietary Sodium-Evoked Up-Regulation of Paraventricular Nucleus Gαi_2_ Proteins to Counter the Development of Salt Sensitive Hypertension

We have previously reported that endogenous up-regulation of PVN Gαi_2_ proteins in response to dietary salt intake is required to for salt resistance in both the Sprague Dawley and Dahl Salt Resistant rat phenotypes (Kapusta et al., 2013; Wainford et al., 2015; Moreira et al., 2019; Carmichael et al., 2020). Our prior studies have demonstrated that the dietary sodium evoked up-regulation of Gαi_2_ proteins, which is conserved across Sprague Dawley and Dahl Salt resistant rats, is specific to the PVN of the hypothalamus with no change observed in the posterior hypothalamus, the supraoptic nucleus or ventrolateral medulla and is specific to Gαi_2_ proteins with no change in the protein expression of Gαi_1_, Gαi_3_ or Gαo proteins in any brain region studied (Kapusta et al., 2013; Wainford et al., 2015; Carmichael et al., 2020). In these studies, we initially replicated our finding that an i.c.v. Gαi_2_ ODN infusion downregulates the protein expression of central Gαi_2_ proteins and evokes the development of the salt sensitivity of blood pressure. At present, the stimulus that evokes the up-regulation of PVN Gαi_2_ proteins in response to dietary salt to remains unknown.

To address this question, we examined blood pressure, PVN Gαi_2_ protein expression, sodium retention and indices of sympathetic tone during normal or high salt intake in animals that underwent an AV3V lesion or ADNX prior to high salt intake. In these studies, animals that underwent a sham or AV3V lesion (verified by adipsia post lesion and absence of pressor response to i.c.v. AngII) exhibited high dietary salt-evoked up-regulation of PVN Gαi_2_ proteins, sympathoinhibition, sodium balance and normotension. In contrast to rats that underwent a sham ADNX procedure, which upregulated PVN Gαi_2_ proteins and remained normotensive, ADNX (verified by reduced renal pelvic CGRP levels) prevented dietary sodium-evoked increase in PVN Gαi_2_ proteins and evoked increased sympathetic outflow and the salt sensitivity of blood pressure. Evidence supporting this is a significant increase in blood pressure during high salt intake, renal sodium retention and increased renal and global sympathetic tone. The magnitude of hypertension observed after ADNX and 21-days high salt-intake was approximately 20 mmHg and is in accord with that observed in our prior studies following central Gαi_2_ protein down-regulation and 21-day high salt intake in Sprague Dawley and Dahl Salt Resistant rats (Kapusta et al., 2013; Wainford et al., 2015). It should be noted that our prior study, Frame et al. (2019) and current work contrast with a prior study, Foss et al. (2015) that suggested the afferent renal nerves have no impact on blood pressure in the Sprague Dawley rat during stepped increases in dietary sodium intake. However, in the prior Foss et al. (2015) study, which concluded 7-weeks post ADNX (1) afferent renal nerve-independent adaptive mechanisms could have been activated to facilitate sodium homeostasis and normotension over the 7-week study period, (2) it is likely physical afferent renal nerve re-innervation occurred but was not functionally assessed, (3) a different experimental paradigm is explored (i.e., stepped increase in dietary sodium vs. ADNX immediately prior to challenge with a high salt intake). Our studies in this, and our prior study Frame et al. (2019) are designed to address the potential confounding effects of non-afferent renal nerve-mediated compensatory mechanisms and functional afferent renal nerve reinnervation. To avoid these potential confounders ADNX was performed immediately prior to the start of high salt intake.

Collectively our current data, which replicate our prior study in Sprague Dawley rats and the Dahl Salt resistant rat in which ADNX immediately prior to high salt intake evoked the salt sensitivity of blood pressure (Frame et al., 2019), suggest that the sympathoinhibitory afferent renal nerve reno-renal reflex is a protective mechanism against the initial increase in blood pressure observed following high salt intake – an observation supported by prior studies following generalized sensory afferent denervation via dorsal rhizotomy or subcutaneous capsaicin treatment in SD rats (Wang et al., 1998, 2001; Kopp et al., 2003). Given that the renal afferent nerves have projections to the PVN our studies suggest that brain Gαi_2_ protein-gated normotensive sympathoinhibitory pathways are upregulated by chronic elevated dietary sodium intake at the level of the kidney via the sensory renal afferent nerves. Based on our prior studies which reported no change in plasma sodium coupled with an increase in urine output to a level that would activate the mechanosensitive afferent renal nerves, (Frame et al., 2019) we speculate it is a mechanosensitive stimulus that evokes up-regulation of PVN Gαi_2_ proteins. Future studies, utilizing retrograde tracing and neuronal phenotyping, will address the potential phenotypes of PVN neurons that express Gαi_2_ proteins that are influenced by activation of the sensory afferent renal nerves.

### Central Gαi_2_ Oligodeoxynucleotide-Mediated Salt Sensitivity of Blood Pressure Occurs Independently of Brain Angiotensin II Type 1 Receptor Signal Transduction

It is well established that activation of the brain AT1R evokes neurogenic hypertension. There is mounting evidence for several shared physiological responses across central AngII-mediated hypertension and our observation of Gαi_2_ protein-dependent salt sensitive hypertension including neuroinflammation and increased sympathetic outflow (Shi et al., 2010; Wainford et al., 2013; Moreira et al., 2019; Carmichael et al., 2020; Mohammed et al., 2020). To investigate the potential interactions between centrally acting AngII and Gαi_2_ subunit protein-gated pathways, we utilized pharmacological antagonism of the AT1R. Evidenced by the fact that verified pharmacological antagonism of the AT1R with losartan did not impact the magnitude of hypertension in Gαi_2_ ODN infused rats on a high salt diet, our data suggest central AT1R activity does not play a role in the development of the salt sensitivity of blood pressure following the down-regulation of brain Gαi_2_ proteins. Collectively, these data support the hypothesis that the salt sensitivity of blood pressure that develops following down-regulation of central Gαi_2_ proteins does not involve a brain AT1R signal transduction mechanism.

## Conclusion

These studies shed light on the interactions of brain Gαi_2_-gated pathways and the sodium/osmosensitive CVOs and sensory afferent renal nerves, sites that influence renal sodium retention and blood pressure. Our data strongly suggest that the central Gαi_2_ gated pathways that regulate blood pressure are activated in part by the sensory afferent renal nerves but are not responsive to central alterations in sodium levels. It appears that central Gαi_2_-gated pathways, which are sympathoinhibitory in nature, are distinct from central salt-activated excitatory pathways that increase blood pressure independently of Gαi_2_ protein signal transduction. Additionally, our data suggest that brain Gαi_2_ protein-gated pathways represent a separate integrated blood pressure regulatory mechanism that is responsive to alterations in whole body sodium homeostasis that functions independently of the brain AT1R. These findings provide new mechanistic insight into the integrated physiological mechanisms that maintain sodium homeostasis and normotension. Given the association of a SNPs in the GNAI2 gene correlating with the salt sensitivity of blood pressure in a sub-set of subjects in the Genetic Epidemiology of Salt Sensitivity dataset, (Zhang et al., 2018) these data suggest pharmacological targeting of the central Gαi_2_ signal transduction system may improve blood pressure control, particularly in a subset of salt sensitive subjects.

## Data Availability Statement

The raw data supporting the conclusions of this article will be made available by the authors, without undue reservation.

## Ethics Statement

The animal study was reviewed and approved by Boston University Institutional Animal Care and Use Committee.

## Author Contributions

JK, CC, JM, and RW performed the experiments. RW prepared the figures. KN, JM, and RW drafted the manuscript. All authors approved final version of manuscript.

## Conflict of Interest

The authors declare that the research was conducted in the absence of any commercial or financial relationships that could be construed as a potential conflict of interest.

## Publisher’s Note

All claims expressed in this article are solely those of the authors and do not necessarily represent those of their affiliated organizations, or those of the publisher, the editors and the reviewers. Any product that may be evaluated in this article, or claim that may be made by its manufacturer, is not guaranteed or endorsed by the publisher.
