# Supplementary figures and images for: Sensory Afferent Renal Nerve Activated Gαi2 Subunit Proteins Mediate the Natriuretic, Sympathoinhibitory and Normotensive Responses to Peripheral Sodium Challenges
Source: Front Physiol. 2021 Nov 30;12:771167. doi: 10.3389/fphys.2021.771167 (PMC8669768; doi:10.3389/fphys.2021.771167)

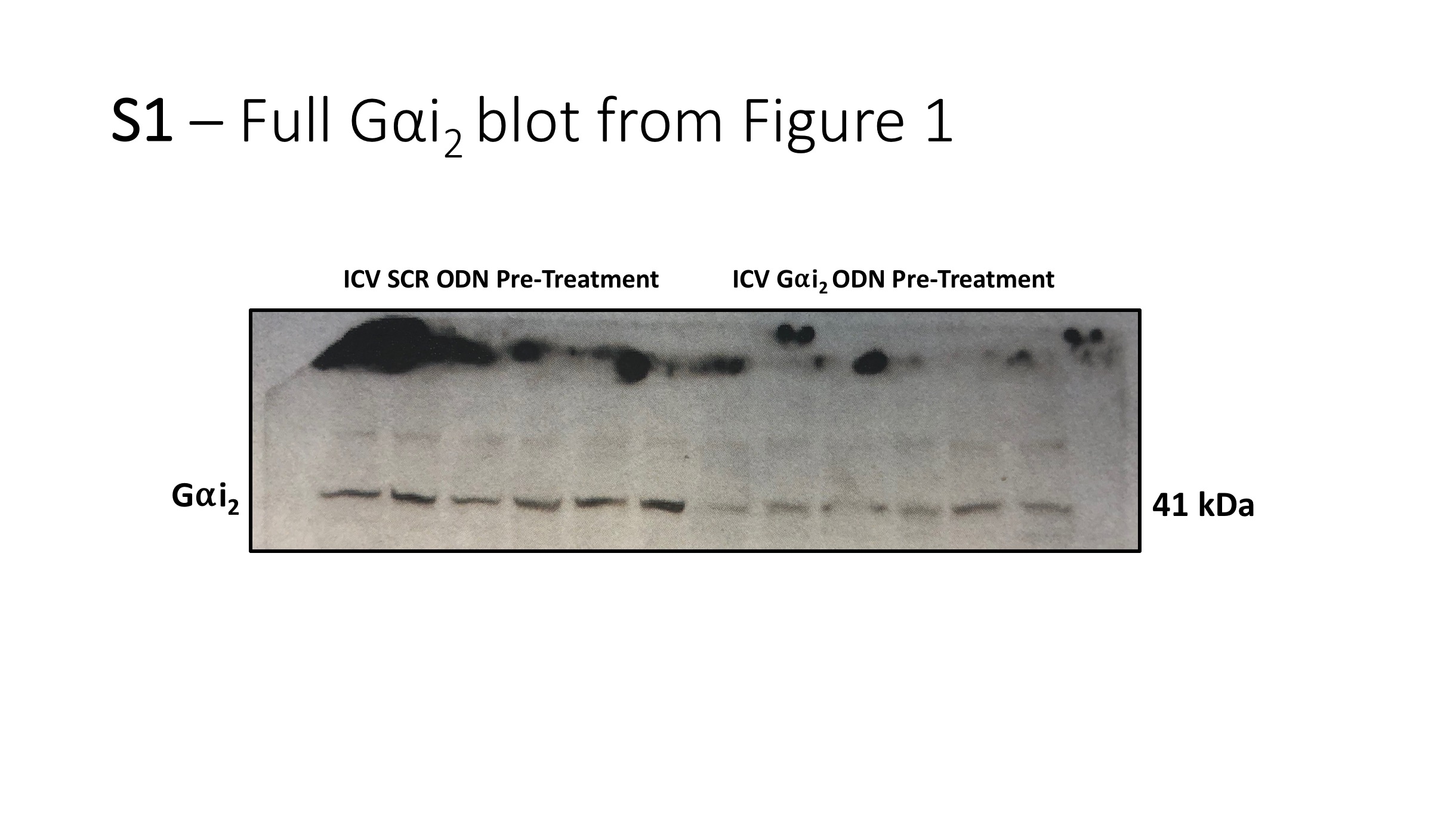

Supplement: Supplementary file 1 [file Image_1.JPEG]

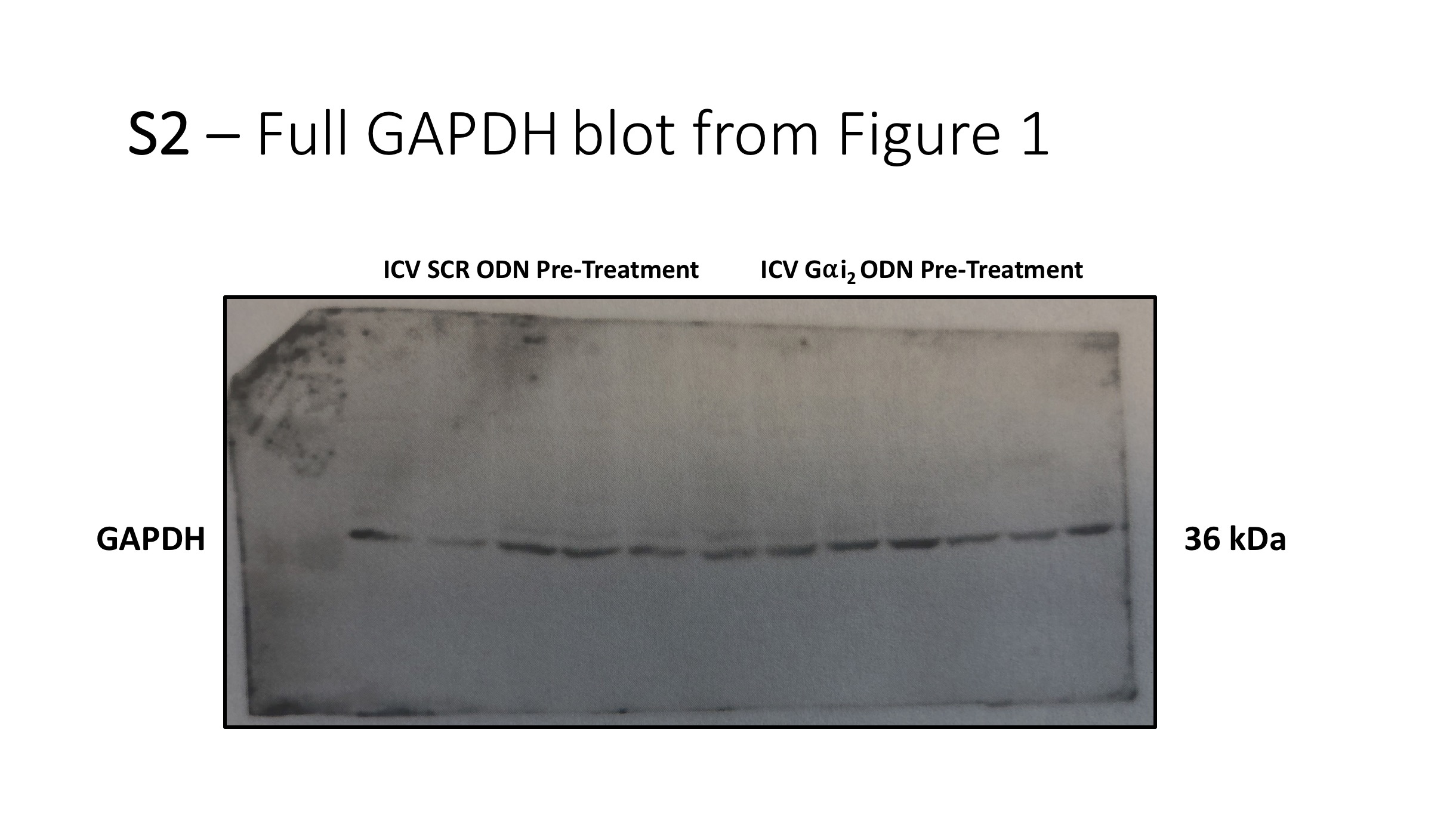

Supplement: Supplementary file 2 [file Image_2.JPEG]

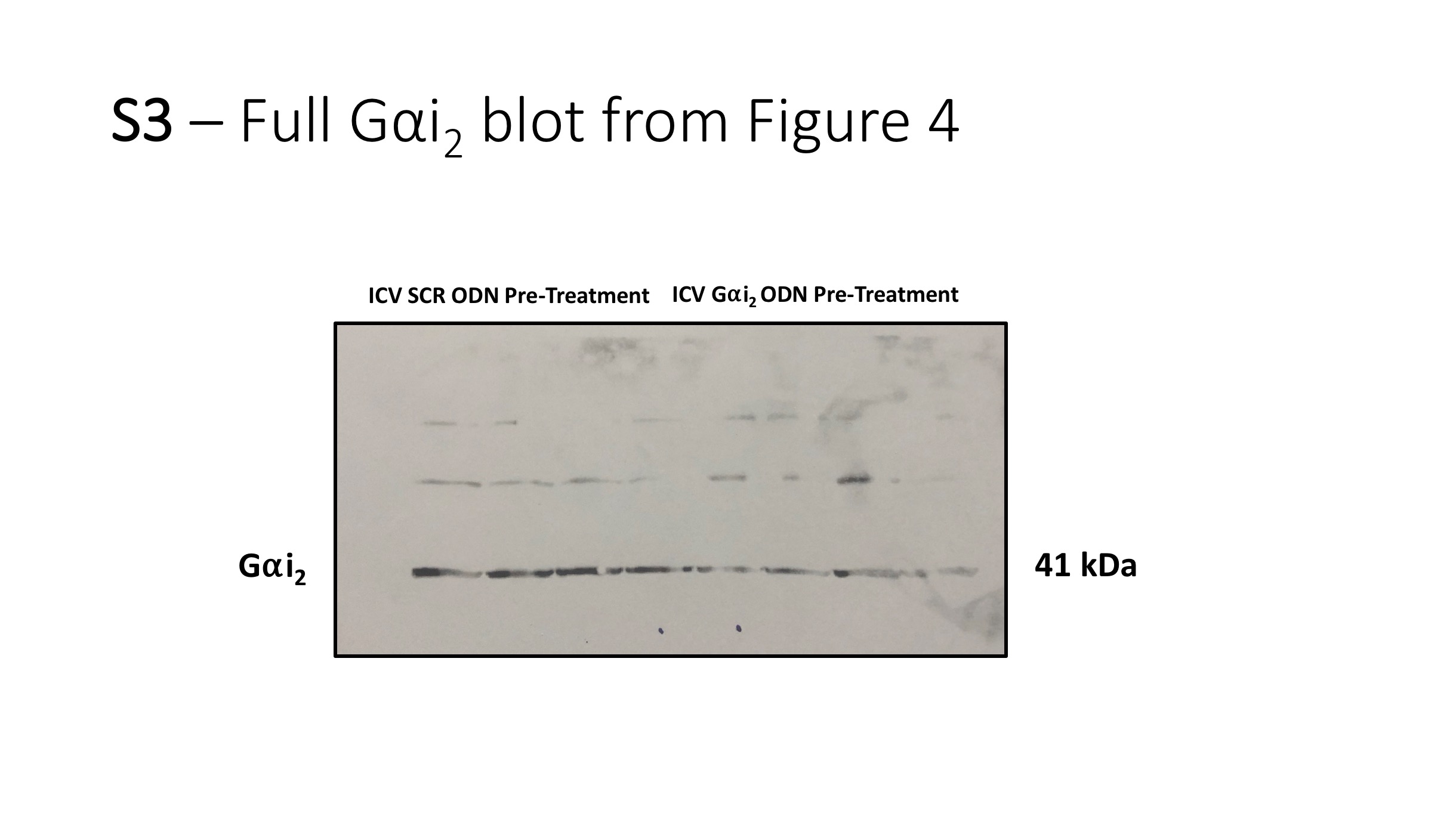

Supplement: Supplementary file 3 [file Image_3.JPEG]

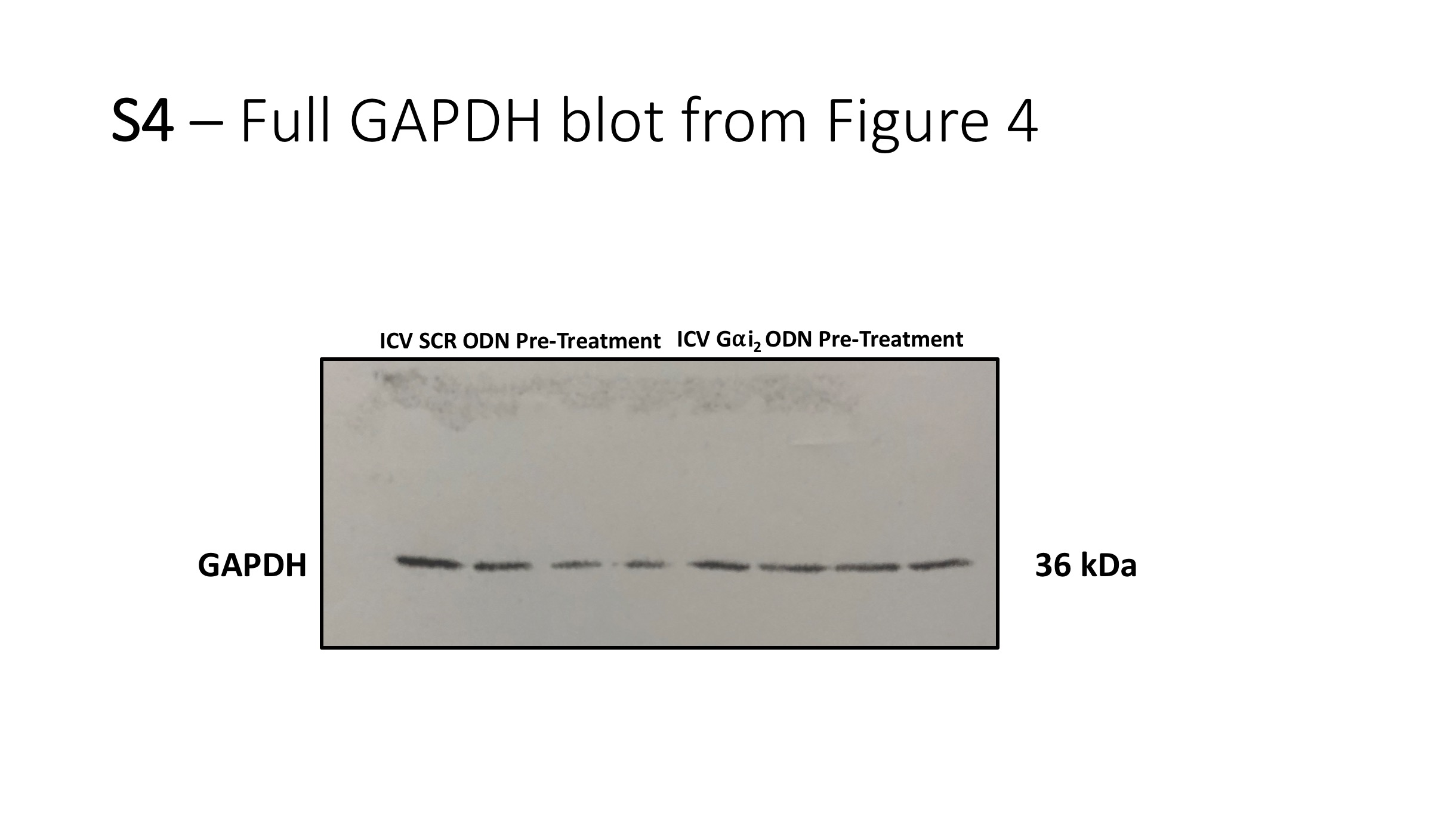

Supplement: Supplementary file 4 [file Image_4.JPEG]

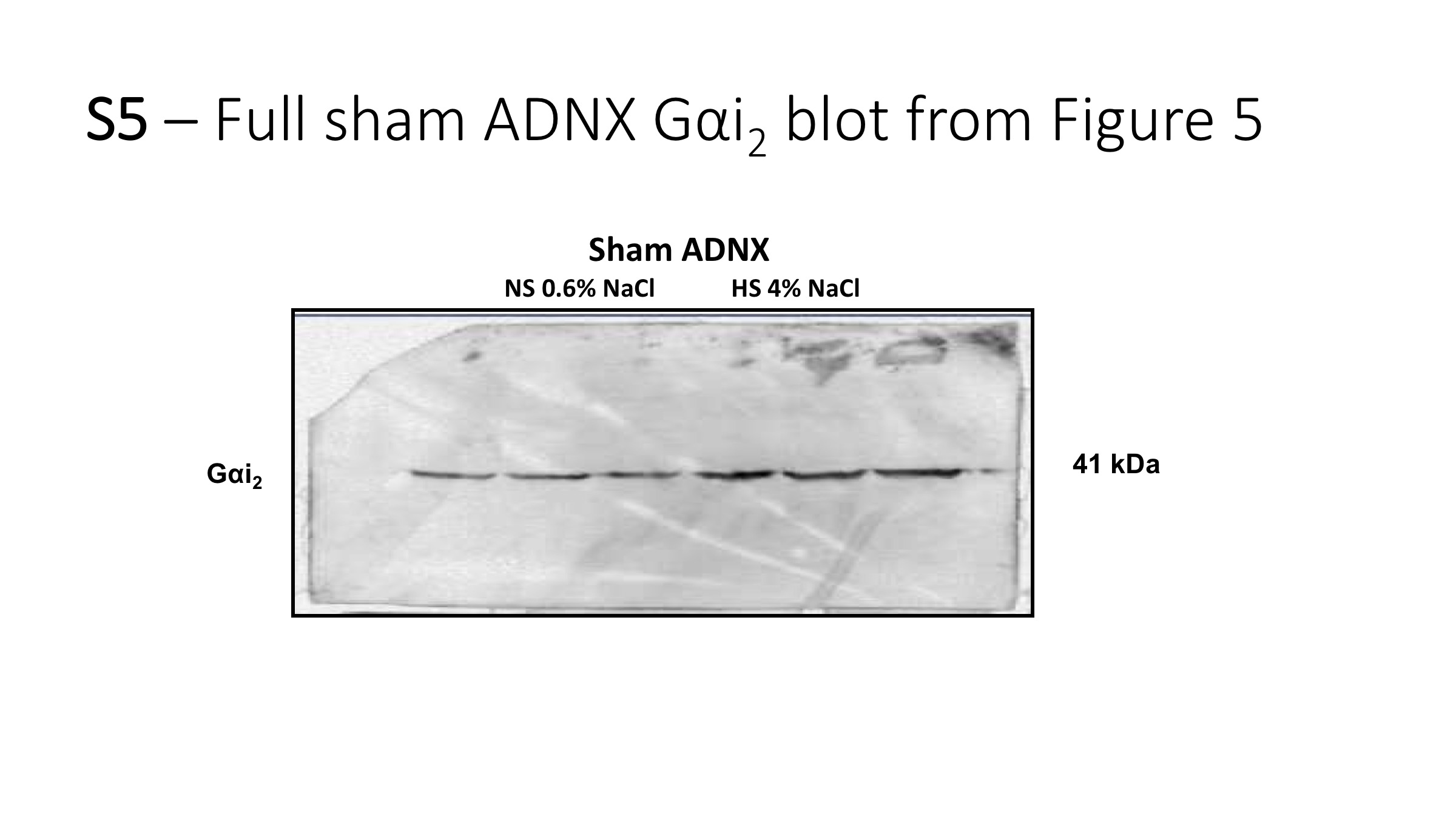

Supplement: Supplementary file 5 [file Image_5.JPEG]

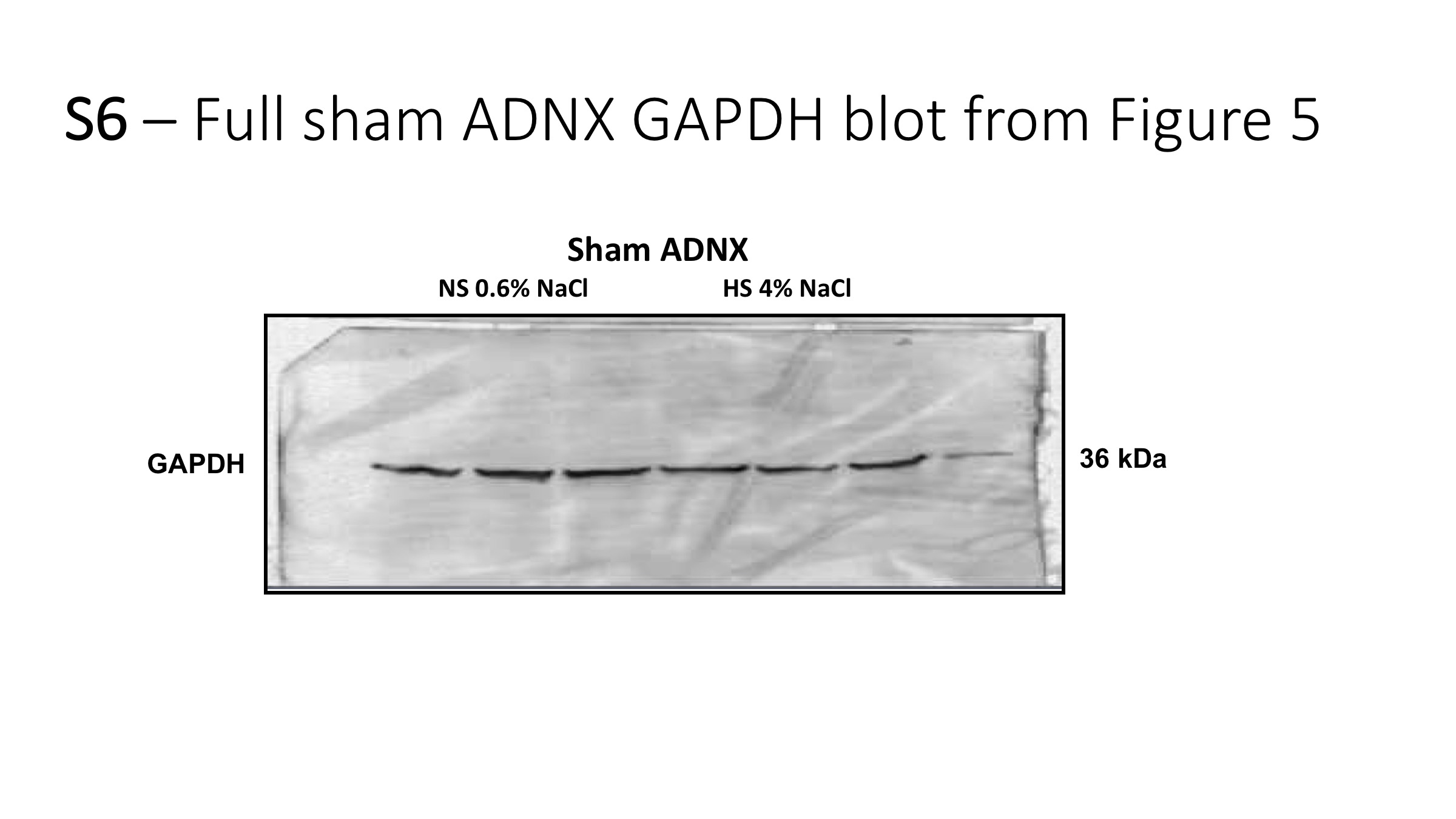

Supplement: Supplementary file 6 [file Image_6.JPEG]

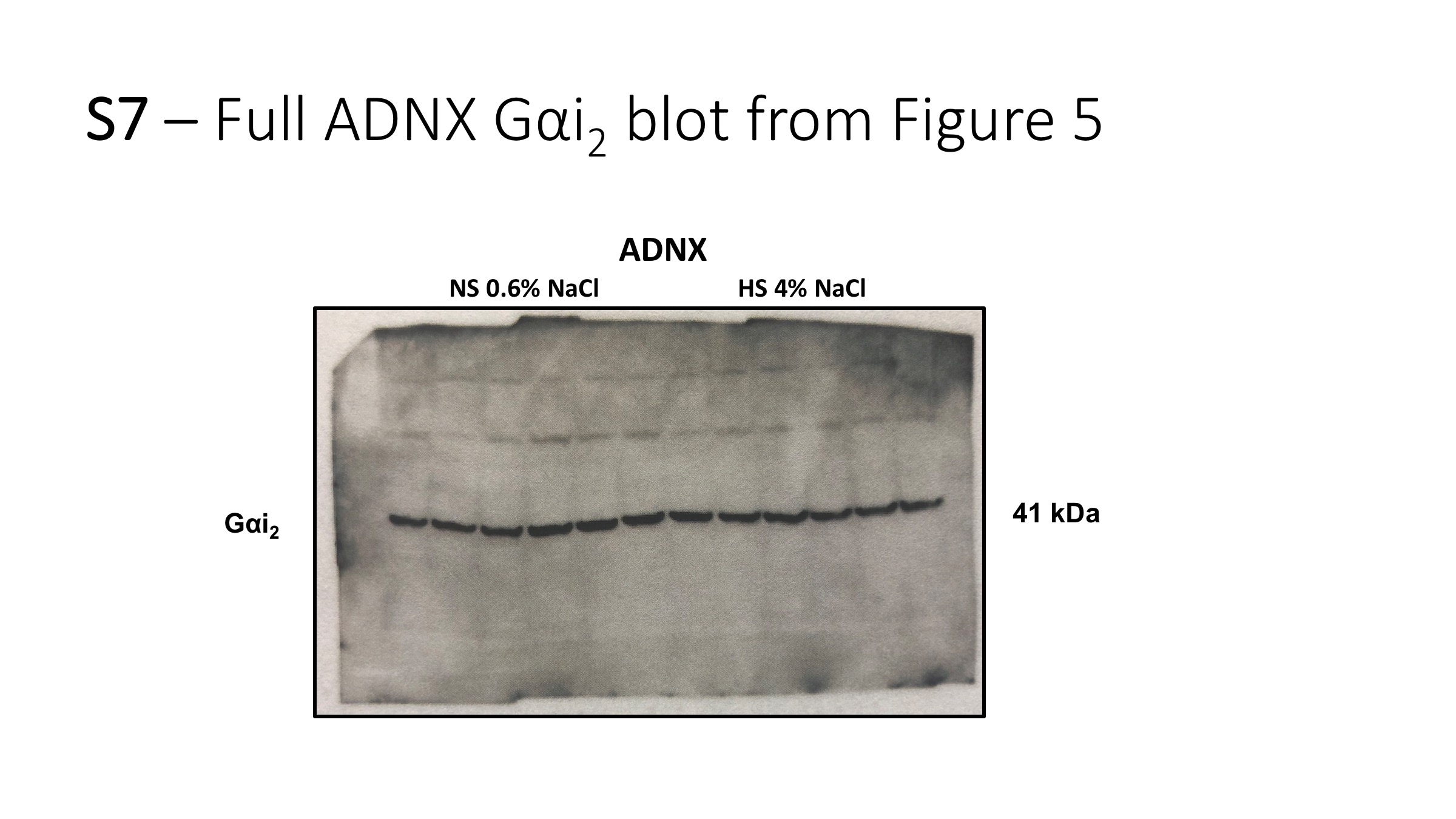

Supplement: Supplementary file 7 [file Image_7.JPEG]

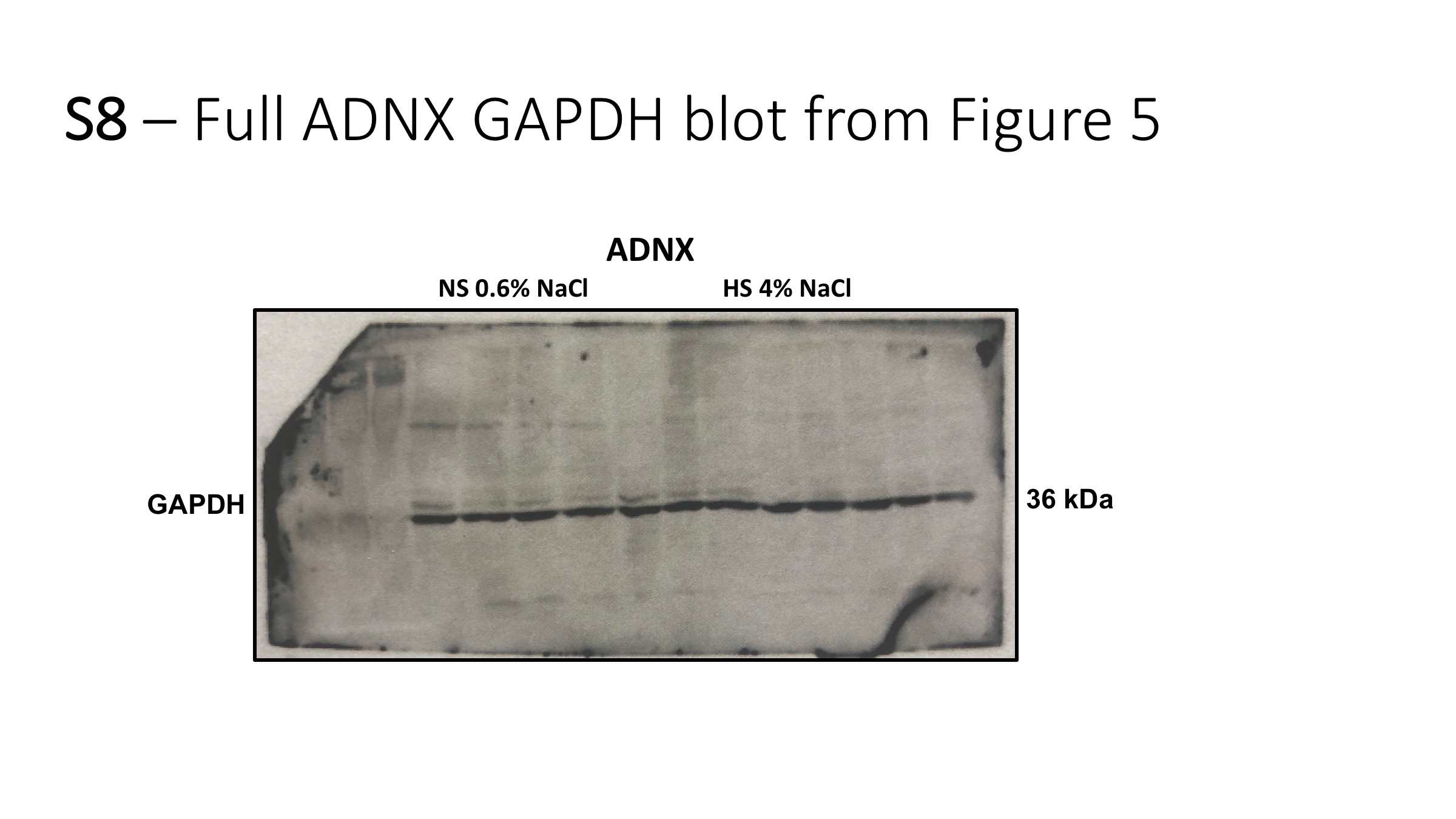

Supplement: Supplementary file 8 [file Image_8.JPEG]

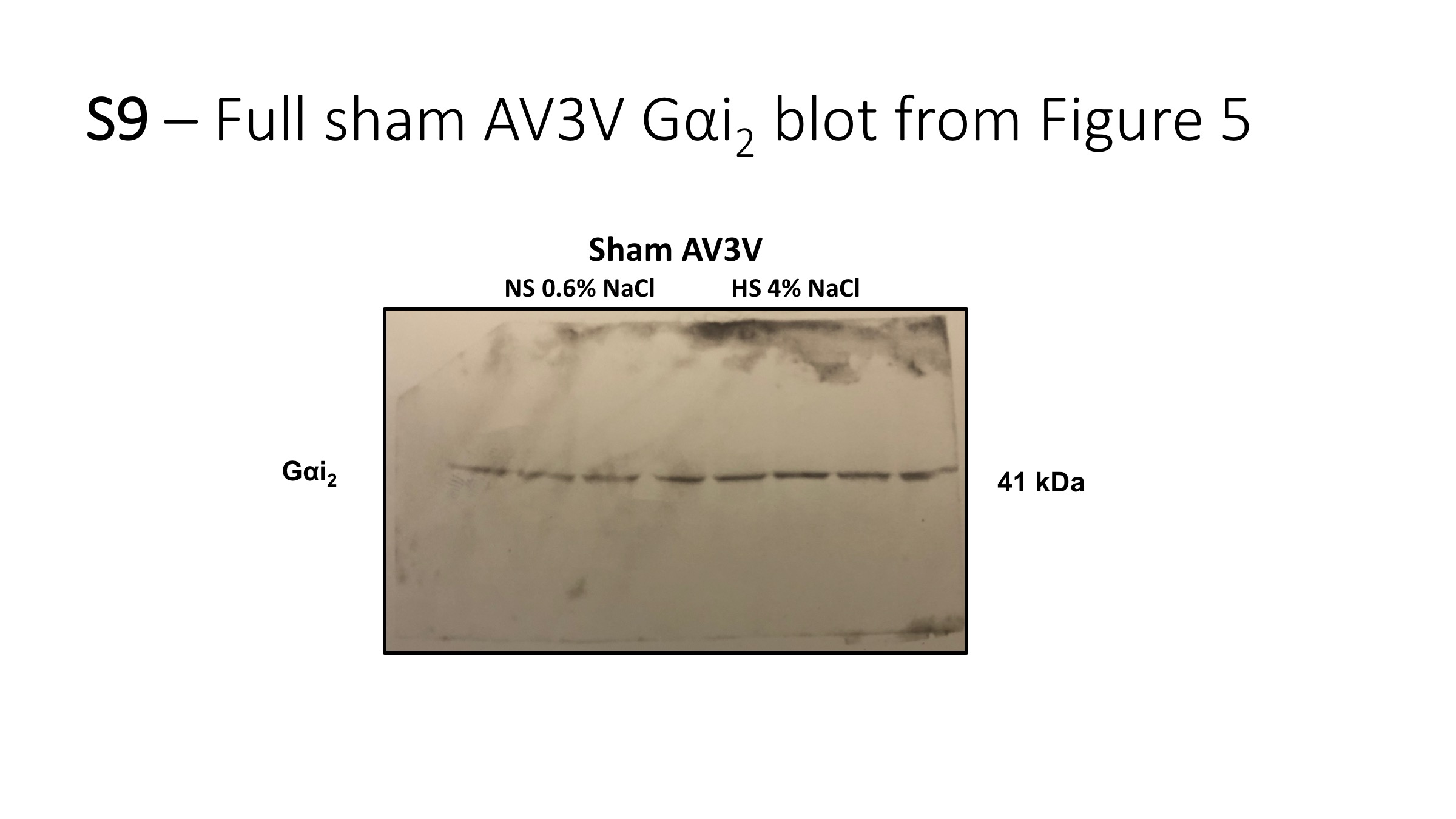

Supplement: Supplementary file 9 [file Image_9.JPEG]

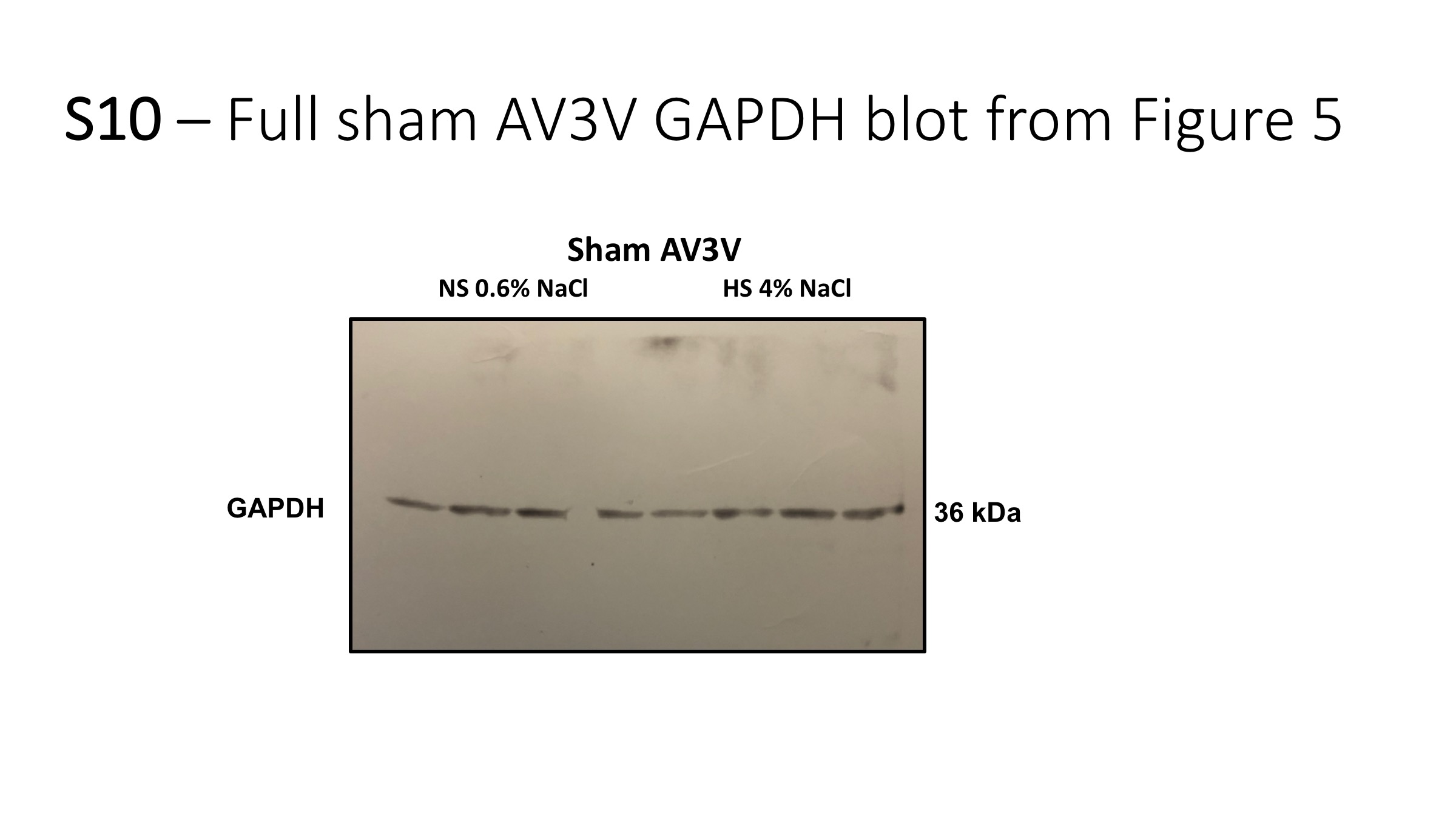

Supplement: Supplementary file 10 [file Image_10.JPEG]

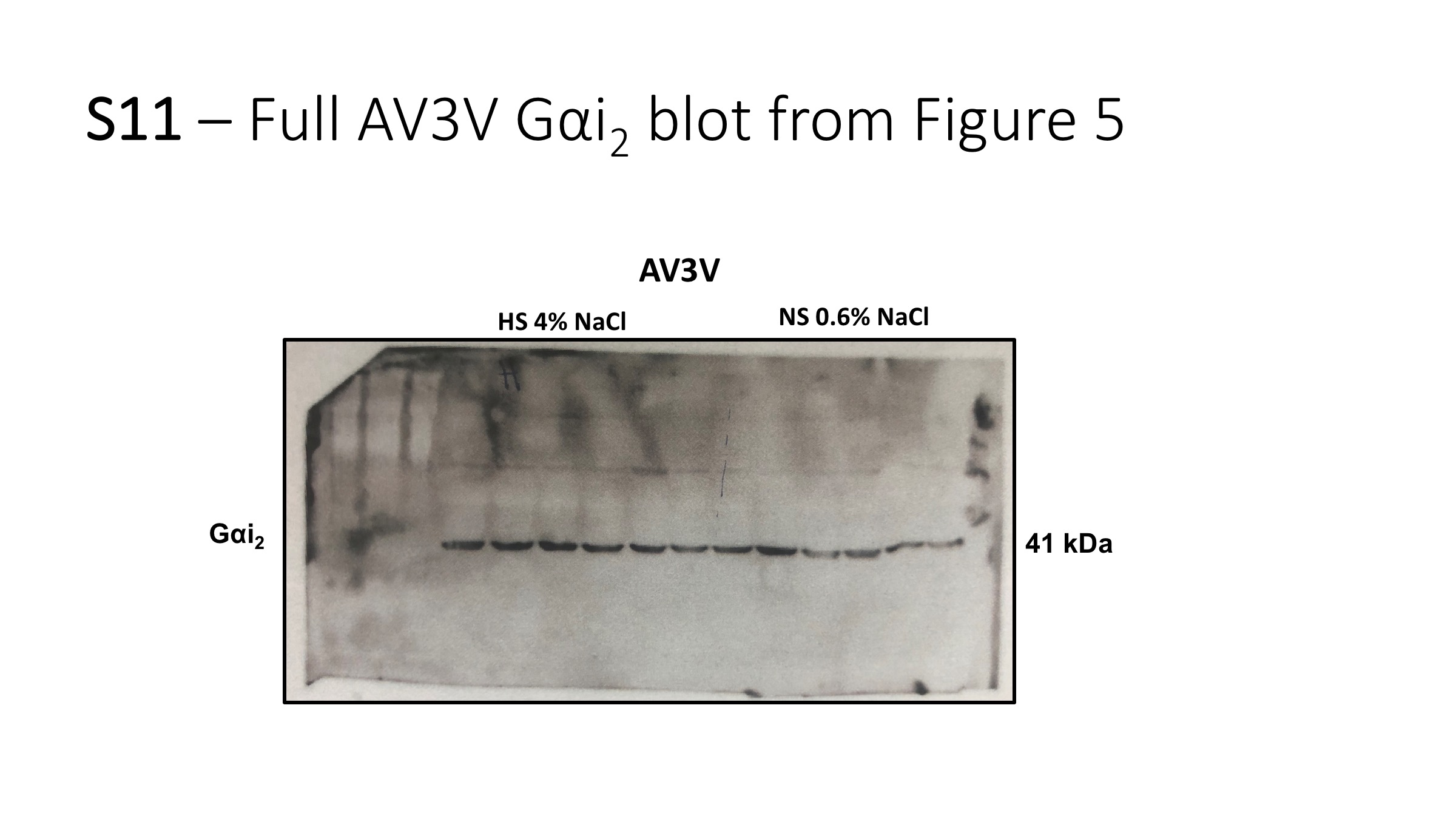

Supplement: Supplementary file 11 [file Image_11.JPEG]

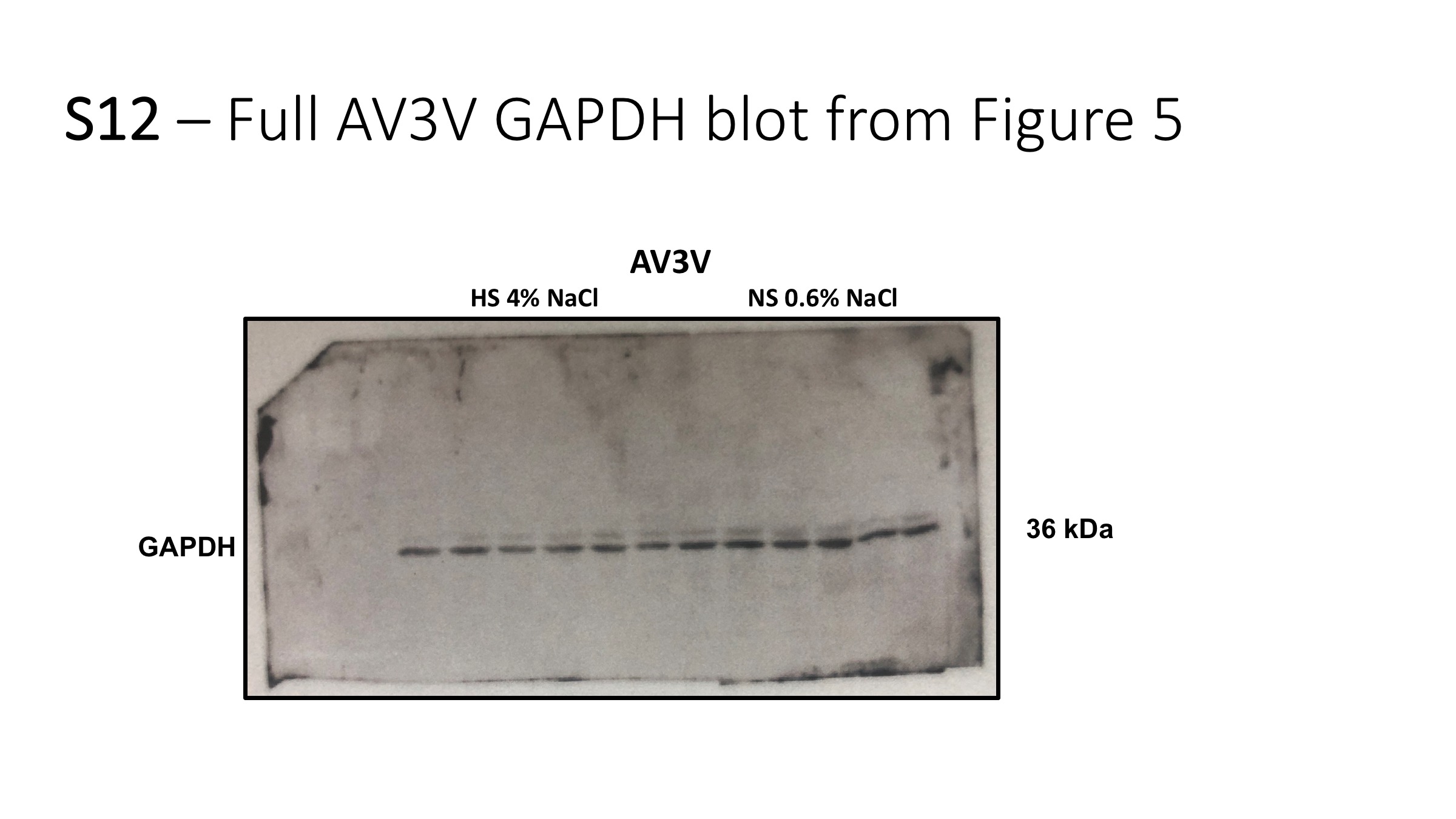

Supplement: Supplementary file 12 [file Image_12.JPEG]
